# In-depth genome and comparative genome analysis of a metal-resistant environmental isolate *Pseudomonas aeruginosa* S-8

**DOI:** 10.3389/fcimb.2025.1511507

**Published:** 2025-02-27

**Authors:** Kiran Kumari, Ayushi Sinha, Parva Kumar Sharma, Rajnish Prakash Singh

**Affiliations:** ^1^ Department of Bioengineering and Biotechnology, Birla Institute of Technology, Ranchi, Jharkhand, India; ^2^ Department of Biotechnology, Jaypee Institute of Information Technology, Noida, India; ^3^ Department of Plant Sciences and Landscape Architecture, University of Maryland, College Park, MD, United States

**Keywords:** bacteria, genome, pan-genome, AMR, virulence

## Abstract

The present study aimed to identify the mechanisms underlying the survival of an environmental bacterium originally isolated from the waste-contaminated soil of Jhiri, Ranchi, India. Based on 16S rRNA, ANI (average nucleotide identity), and BLAST Ring Image Generator (BRIG) analysis, the isolated strain was identified as *Pseudomonas aeruginosa.* The present study extends the characterization of this bacterium through genomic and comparative genomic analysis to understand the genomic features pertaining to survival in stressed environments. The sequencing of the bacterium at Illumina HiSeq platform revealed that it possessed a 6.8 Mb circular chromosome with 65.9% GC content and 63 RNAs sequence. The genome also harbored several genes associated to plant growth promotion *i.e.* phytohormone and siderophore production, phosphate solubilization, motility, and biofilm formation, etc. The genomic analysis with online tools unraveled the various genes belonging to the bacterial secretion system, antibiotic resistance, virulence, and efflux pumps, etc. The presence of biosynthetic gene clusters (BCGs) indicated that large numbers of genes were associated to non-ribosomal synthesized peptide synthetase, polyketide synthetase, and other secondary metabolite production. Additionally, its genomes encode various CAZymes such as glycoside hydrolases and other genes associated with lignocellulose breakdown, suggesting that strain S-8 have strong biomass degradation potential. Furthermore, pan-genome analysis based on a comparison of whole genomes showed that core genome represented the largest part of the gene pools. Therefore, genome and comparative genome analysis of *Pseudomonas* strains is valuable for understanding the mechanism of resistance to metal stress, genome evolution, HGT events, and therefore, opens a new perspective to exploit a newly isolated bacterium for biotechnological applications.

## Introduction

*Pseudomonas* genus is considered a major opportunistic pathogen, isolated from diverse ecological niches including, soil, water, and clinical specimens (Stover et al., 2000), and possess intrinsically advanced antibiotic resistance gene clusters (Lambert, 2002). Many strains have been associated with serious illness-causing nosocomial infections and various sepsis syndromes (Planquette et al., 2013; Philippart et al., 2015). The pathogenicity is attributed to the presence of virulence features like pili, flagella, exotoxin A, secretion system, and quorum-sensing proteins (Lyczak et al., 2000). Among various members, *P. aeruginosa* is a Gram-negative, aerobic, rod-shaped bacterium and environmental isolates of this bacterium easily adapt to a large variety of natural ecosystems (Selezska et al., 2012). As every *P. aeruginosa* has a certain pathogenic potential, they are all classified as risk group two and the degree of virulence varies substantially between strains (Hilker et al., 2015).

Besides, soil-dwelling *Pseudomonas* species form close relationships with plants, thereby positively affecting plant growth and nutrition (Cheng et al., 2017; Zamioudis et al., 2013), and also exhibits potent antagonistic activity toward pathogenic microorganisms (Biessy et al., 2019). However, the infection caused by various phytopathogens leads to a loss of ~25% crop production globally (Kim et al., 2017a, b). Additionally, the excessive use of chemical pesticides and fertilizers used to control phytopathogens imposes a serious effect on human and environmental health (Alvarez et al., 2012). Under these circumstances, microbial inoculants belonging to the genera *Pseudomonas* holds a promising substitute for conventional fertilizers and antibiotics (Stewart, 2001). *Pseudomonas* spp. influence plant health also by the production of diverse set of secondary metabolites (Nguyen et al., 2016; Stringlis et al., 2018), and through secreted proteins (Rangel et al., 2016). The production of phenazines and anthranilate by the *P. aeruginosa* contribute to the antagonism against plant pathogens (Anjaiah et al., 1998), and is thereby used as biocontrol agents and biofertilizers (Kwak and Weller, 2013). Moreover, *Pseudomonas* spp. also employ diverse mechanisms to colonize the plant rhizosphere (Little et al., 2019) and suppress a range of plant pathogens including bacteria (Arseneault et al., 2015), fungi (Michelsen et al., 2015), and insects (Flury et al., 2017), however, these attributes vary from strain to strain.

The genome of *Pseudomonas* spp. generally divided into a core genome containing sequences common to the species and an accessory genome, with the restriction of sequences present in some strains (Kung et al., 2010; Ozer et al., 2014). The virulence of *Pseudomonas* spp. is generally affected by the variation in both the core and accessory genome. However, many of the genomes investigation are still going, therefore, accessory genes associated with virulence in addition to mutation may be missed. This may limit the prediction of antibiotic resistance or virulence genes on mobile genetic elements (MGE) or standard chromosome regions as well as their prevalence in general (Beatson and Walker, 2014). It was suggested that the large genome size and complexity of several *P. aeruginosa* strains such as *P. aeruginosa* PAO1 (6.2 Mbp, 5683 genes), *P. aeruginosa* PA14 (6.5 Mbp, 5965 genes), and *P. aeruginosa* PA7 (6.5 Mbp, 6369 genes) reflects environmental adaptation with the highest proportion of regulatory genes, and a large number of genes involved in metabolism, transport and efflux systems allows the bacterium to survive in diverse environments (Stover et al., 2000; Winsor et al., 2011). The higher number of regulatory genes modulates the metabolic and genetic capacity of *P. aeruginosa* in varying environmental conditions. The increased availability of genes, genomics data, and further use of genomic tools suggests the potential applications of the strain for diverse biotechnological applications (Xia et al., 2018). Many of the *Pseudomonas* strains possess good industrial applications in-terms of production of highly stable enzymes (Grbavčić et al., 2011), biosurfactants (Rikalović, 2013), and polysaccharides (Dimitrijević et al., 2011). Additionally, many of the strains have been reported to tolerate high concentrations of heavy metals (Izrael-Zivkovic et al., 2018), which makes it industrial useful.

Due to the spectrum of ecological, biochemical and metabolic characteristics of the *Pseudomonas* genus, it is clearly evident that diversity among this bacterium extends to the genomic level. Klockgether et al. (2011) have suggested that sequencing of *P. aeruginosa* strains from environmental habitats provides an unbiased overview of the genetic repertoire. More than 200 P*. aeruginosa* genome sequences are available on the National Centre of Biotechnology Information (NCBI), however, less than 10% are of environmental strains. The increase in robust sequencing technologies has resulted in the economic cost of sequencing a bacterial genome. Next-generation sequencing provides valuable insight into the genome of organisms and allows the comprehensive analysis of genomic features (Roy et al., 2013). Furthermore, the functional annotation of genomic features can be utilized as a powerful tool for the development of genetically modified bacteria with improved functionality. Additionally, comparative genomics has emerged as a robust tool to identify and compare functionally important genomic elements (Wu et al., 2010). A comparison of genomes within the *Pseudomonas* group demonstrated evidence about the ecological and physiological diversity of these bacteria extends to the genomic level (Loper et al., 2012).

In the present study, we focused on the detailed genomic characterization of environmental isolate *P. aeruginosa* S-8 isolated from waste-dumping soil sample, which showed good antagonistic activity against tested bacterial and fungal pathogens. Therefore, the in-depth genome and comparative genome analysis will fill the gap in the genomic studies of environmental *Pseudomonas* strains. Additionally, the whole-genome analysis (WGS) of this strain will provide opportunities to identify genes involved in the biocontrol of pathogens, plant growth promotion (PGP), and genes involved in the production of secondary metabolites, etc. The available WGS data of many *Pseudomonas* strains in the public database (https://www.ncbi.nlm.nih.gov/datasets/genome/) have been used for the comparative genome analysis and pan-genome analysis with WGS data of *P. aeruginosa* S-8.

## Materials and methods

### Characterization of strain S-8

The bacterial strain S-8 was isolated from the metal-contaminated soil of Jhiri (23.40°N, 85.25°E), Ranchi, Jharkhand, India and its genome was submitted with accession number JARESC000000000. However, before genome sequencing, we performed the 16S rRNA gene sequencing following standard protocol (Singh et al., 2024). The strain was tested for its metal-stress tolerance against zinc sulfate (ZnSO_4_), copper sulfate (CuSO_4_), cadmium chloride (CdCl_2_), mercuric chloride (HgCl_2_), and nickel sulfate hexahydrate (NiSO_4_.6H_2_O), each with 5 mM concentration (Kumari et al., 2024). The isolate was tested for antagonistic activity against bacterial pathogens such as *Bacillus subtilis, Salmonella typhi, Escherichia coli*, and *Staphylococcus aureus* by using a well-diffusion method. The boiled culture was used as a control and the experiment was performed in triplicate. To test the antifungal activity, 100 µl fungal mycelia (in 0.85% saline) of *Aspergillus niger, Microsporum gypseum, H. gypsium* and *Penicillium citrium* was spread on the potato dextrose agar (PDA, Himedia, India) plate, and wells of 6 mm diameter were filled with 1×10^8^ CFU/ml of S-8, and kept for incubation at 28°C for seven days. The activity was evaluated by measuring the ZOI (zone of inhibition) for which the parameter used was <10 mm = poor (+), 11 to 15 mm moderate (++), and between 16 to 20 mm = good (+++).

Isolate S-8 was tested for various motility behavior. To perform the swimming, S-8 was spot-inoculated on solidified tryptone swim plate composed of 1% tryptone, 0.5% NaCl, 0.3% agar, and incubated for 16 hr at 25°C. To evaluate the swarming, S-8 was spot inoculated on media containing 0.5% bacto-agar, 8g L^-1^ nutrient broth, 5g L^-1^ dextrose, and incubated for 24 hr at 30°C. For twitching, LB agar (1% agar) media was used for stab inoculation, and inoculated plates were incubated at 30°C for 24-48 hr. After incubation, the presence of a turbid circular zone indicated the swimming, movement of inoculation for swarming, and circular turbid zone for twitching activity (Connelly et al., 2004). The experiment was performed in duplicate.

### Antibiotics sensitivity test

To check the antibiotics susceptibility, the disk diffusion method was used as recommended by the Clinical and Laboratory Standards Institute (CLSI). The isolate was grown in LB broth overnight for 14h with shaking at 180 rpm, spread in an agar plate, and then the paper disk containing the different antibiotics such as erythromycin (15 µg), ampicillin (10 µg), kanamycin (30 µg), tetracycline (30 µg), ciprofloxacin (5 µg), gentamicin (10 µg), fluconazole (25 µg), streptomycin (10 µg), vancomicin (30 µg), and voriconazole (10 µg) was placed in the agar plate for diffusion and incubated for 24-48 hr. The result was observed by measuring the diameter of the zone of inhibition created by the antibiotic disk against the tested isolates. These are the most common antibiotics used against both environmental and clinical strains (Singh et al., 2024).

### Whole genome sequencing

The extracted genomic DNA was sequenced using an illumina HiSeq platform and the paired-end library was prepared by using the NEB Next Ultra DNA Library Prep Kit. Fast QC program was used for Quality control of Illumina reads (http://www.bioinformatics.babraham.ac.uk/projects/fastqc). The Illumina reads were assembled using genome assembly tools SPAdes (Nurk et al., 2013). The transfer RNA (tRNA) and ribosomal RNA (rRNA) of the S-12 strain were identified using the tRNAscan-SE and RNAmmer (v1.2, http://www.cbs.dtu.dk/services/RNAmmer/) software, respectively. Further, the genome sequence was annotated by RAST (Rapid Annotation using Subsystem Technology) annotation server (Aziz et al., 2008) to annotate the open reading frames, and BLAT v2.0 to validate the predictions. COG functions of protein-coding sequences were determined using the RPS-BLAST algorithm for blast search against the COG database (https://ftp.ncbi.nih.gov/pub/wolf/COGs/) (Schaffer et al., 2001; Deb, 2022). The genes involved in metabolic pathways were annotated using KEGG and Blast2Go tools. Using Genome BLAST Distance Phylogeny (GBDP) method and tree builder service, the phylogeny tree of S-8 was created using its whole genome sequence.

### Average nucleotide identity analysis

ANI analysis was done to explore the genetic distance and relatedness for the genome sets containing S-8, and publicly available *P. aeruginosa* genomes (**Table 1**) by MASH v2.2.2 (Ondov et al., 2016). To improve the speed for analyzing large amounts of sequence data, other methods have been developed including FastANIv1.32 which uses an alignment-free mapping algorithm (Mashmap) implemented to approximate ANI calculations in a range of 80–100% identity (Jain et al., 2018).

**Table 1:** General features of *P. aeruginosa* S-8 genome.

Property	Term
Geographical location	23.40°N, 85.25°E
Sample collection	Soil
NCBI Bioproject ID	PRJNA934691
Bio-Sample ID	SAMN33277316
GenBank ID	JARESC000000000
Sequencing platform	Illumina HiSeq 2000
Genome size	6.3 Mb
G+C Content	65.9%
N50	243357
L50	8
Contig no	1
Protein-coding genes	6,398
RNAs genes	63

### Antimicrobial and virulence analysis

The CARD database was used using a homology-based approach (BLASTX) against the genome sequence of S-8 to unravel the presence of AMR genes. For searching, BLAST output was filtered with a minimum of 80% identity and subject protein coverage. Similarly, the VFDB database was used against assembled genome with criteria of a minimum of 80% identity using a homology-based approach (BLASTX) to identify the virulence genes.

### Prediction of biosynthetic gene clusters

The number and types of secondary metabolite BGCs in the genome sequence of *P. aeruginosa* S-8 were identified by antiSMASH version 5.1.2 in combination with Hidden Markov Model (HMM) to detect the BGCs-like region (Blin et al., 2021). Various unknown and characterized BGCs were identified and genetic similarities in gene clusters were predicted using antiSMASH 5.1.2.

### Prediction of carbohydrate−active enzyme (CAZymes)

To unravel the presence of various CAZymes including glycosyltransferases (GTs), glycoside hydrolases (GHs), polysaccharide lyases (PLs), carbohydrate esterases (CEs), auxiliary activities (AAs) and carbohydrate-binding modules (CBMs), the protein sequences of S-8 was annotated using the dbCAN2 server, and BLAST-driven DIAMOND against the CAZy database. The diversity of CAZymes in the closest relatives of *P. aeruginosa* strains, *P. aeruginosa* NCTC9433, *P. aeruginosa* SCV20265, *P. aeruginosa* PA_D2, *P. aeruginosa* W36662, *P. aeruginosa* Paer4_119, *P. aeruginosa* F30658, *P. aeruginosa* PASGNDM345, *P. aeruginosa* PA1R, *P. aeruginosa* X78812, and *P. aeruginosa* W16407 was also performed to evaluate the comparative distribution.

### Comparative genome analysis

The distribution of genes in the selected genome (**Supplementary Table 1**) under different functional category like virulence, metabolism, carbohydrate, stress responses, protein metabolism, amino acids & derivatives, and membrane transport was performed by RAST analysis. The analysis of orthologous gene clusters was analyzed using the Orthovenn2 program (Xu et al., 2019) with default parameters using the protein sequences of *P. aeruginosa* S-8, *P. aeruginosa* NCTC9433, *P. aeruginosa* SCV20265, *P. aeruginosa* PA_D2, *P. aeruginosa* W36662, and *P. aeruginosa* Paer4_119. The genomes were selected based on the high similarity to S-8 strains. The circular genome comparison of the assembly genome of S-8 was performed against the reference genomes (**Supplementary Table 1**) using the BRIG (Blast Ring Image Generator) Tool (Alikhan et al., 2011). BLAST was performed on five characteristic *Pseudomonas* genomes which were constructed using NCBI local BLAST-2.10.1.

### Pan-genome analysis

Core and accessory genes in *P. aeruginosa* S-8 and its closest related strains were identified by using Roary 3.11.2 with default settings. The GFF3 files of all selected strains including *P. aeruginosa* S-8 genomes were generated by PROKKA 1.14.5 (Seemann, 2014). The maximum likelihood (ML) phylogenetic tree of *P. aeruginosa* S-8 and its closest related strains based on core-genome single nucleotide polymorphism was plotted after filtering the core genome alignment using the SNP-sites 2.5.1 (https://github.com/sanger-pathogens/snp-sites) (Page et al., 2016). The evolutionary history was calculated by the ML method based on the Tamura and Nei method (Tamura and Nei, 1993). To visualize the matrix showing the presence and absence of core genes in the used strains, Phandango was used. The summary file generated by Roary software was used to assess the proportions of the pan-genome.

### HGT and SNP analysis

HGTector2 bioinformatics tool was used for the detection and analysis of horizontal gene transfer (HGT) events in microbial genomes. It utilizes computational methods to identify genes or genomic regions that have likely been acquired through horizontal transfer from distantly related species. Here, we used all the genomes as input for HGT analysis. Parsnp v.1.2 was used for whole-genome alignment and phylogenetic analysis of microbial genomes (Treangen et al., 2014). It compares genomes to a reference (using MUMmer) to identify core genome SNPs and build a phylogeny. It is part of the Harvest suite of tools developed by the University of Maryland, School of Medicine. The main purpose of Parsnp is to align and compare multiple bacterial genomes to identify genetic variations and to infer the phylogenetic relationships. It employs a progressive Mauve algorithm, which is a multiple genome alignment method that takes into account genome rearrangements, inversions, and horizontal gene transfer events.

### Data deposition

The genome sequence of S-8 is available at NCBI with the BioProject PRJNA934691, Biosample SAMN33277316, and genome accession no. JARESC000000000.

## Results

### Characterization of strain S-8

**Figure 1:**
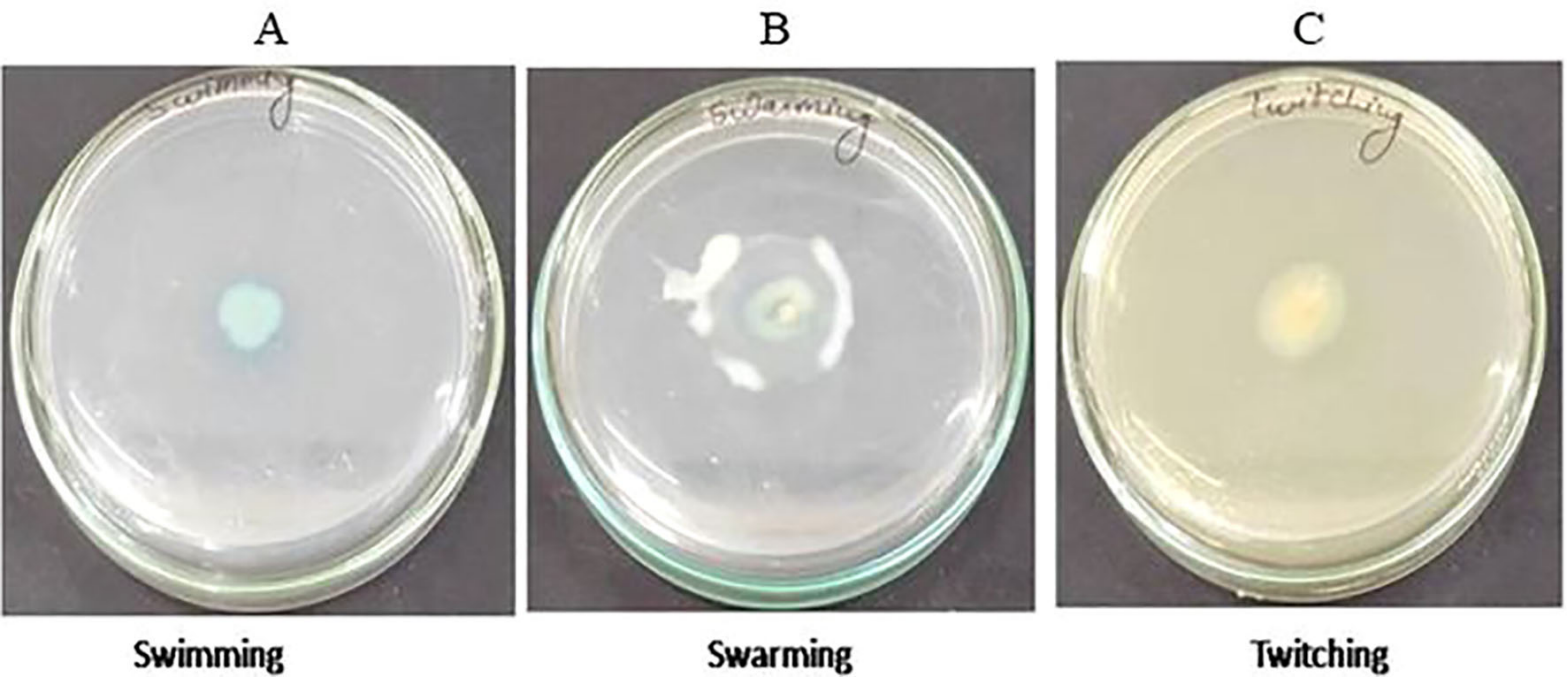
Test of motility swimming **(A)**, swarming **(B)**, and twitching **(C)** shown by *P. aeruginosa* S-8 on motility specific medium plate.

The 16S rRNA sequencing confirmed that isolated strain S-8 belongs to *P. aeruginosa* (**Supplementary Figure 1**). The growth pattern of S-8 in metal enriched medium showed that strain S-8 growth behavior was higher in CuSO_4_-amended medium as compared to other heavy metals. The isolate showed a higher sensitivity (20 to 25 mm) against ampicillin, kanamycin, tetracycline, gentamicin, ciprofloxacine, vancomycin, and moderate sensitivity (12 to 18 mm) to streptomycin, and fluconazole. The strain was found to be resistant to voriconazole and erythromycin. The test isolate showed good antagonistic activity against *S. typhi, E. coli*, and moderate against *B. subtilis* and *S. aureus.* Against the tested fungal strains, S-8 showed good activity against *A. niger, M. gypsium*, and moderate against *H. gypsium, P. citrium* (**Supplementary Figure 2**). The test isolate S-8 showed the swimming, swarming, and twitching motility (**Figure 1**).

### Genome analysis

**Figure 2:**
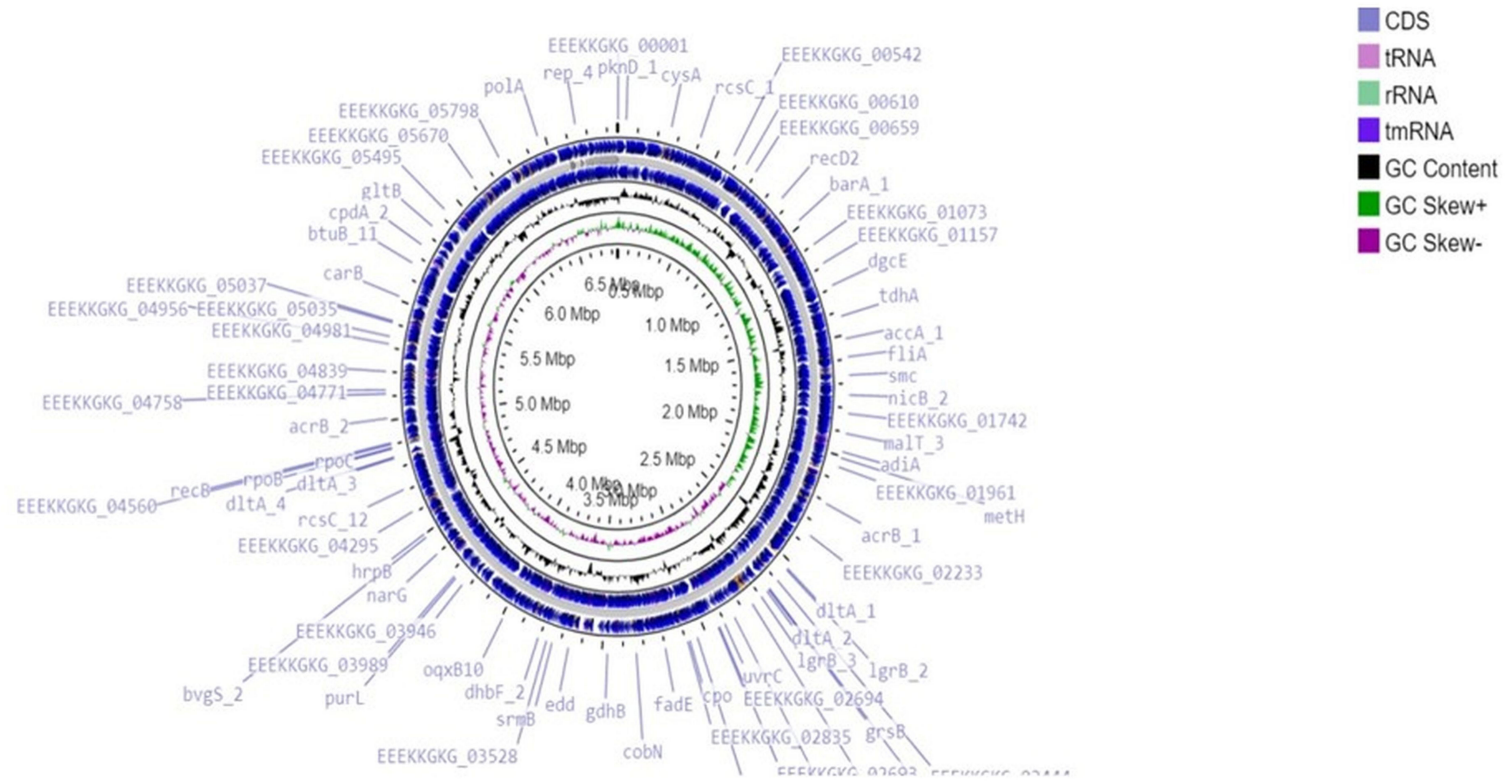
The circular genome map (6.8 Mbp) of *P. aeruginosa* S-8. From the outer circle to the inner circle: coding DNA sequences (CDS) on the forward and reverse strand, RNA (tRNA/rRNA), GC content and GC skew, Pie chart representing the RAST subsystems categories in the *P. aeruginosa* S-8 genome. The most abundant systems on the category level are shown in the left piet chart, whereas, the right column showing the counts of features.

A total of 6,908,234 sequencing reads were generated for strain S-8. The assembly of the genome sequence was performed using the Unicycler v-0.48 tool and a single contig of 6.8 Mb size was obtained (**Figure 2**). The assembly was validated using the NCBI-NR Blast program which showed the maximum homology with other *Pseudomonas* spp. The overall average G+C content of *P. aeruginosa* S-8 is 65.9% (**Table 1**) and therefore considered as G+C-rich, whereas genes acquired through horizontal gene transfer usually have a lower G+C content. A total of 63 RNAs were annotated in the S-8 genome. Further, gene/protein prediction from the draft genome using the Prokkav1.14 tool identified a total of 6,398 protein-coding genes.

### RAST functional annotation

**Figure 3:**
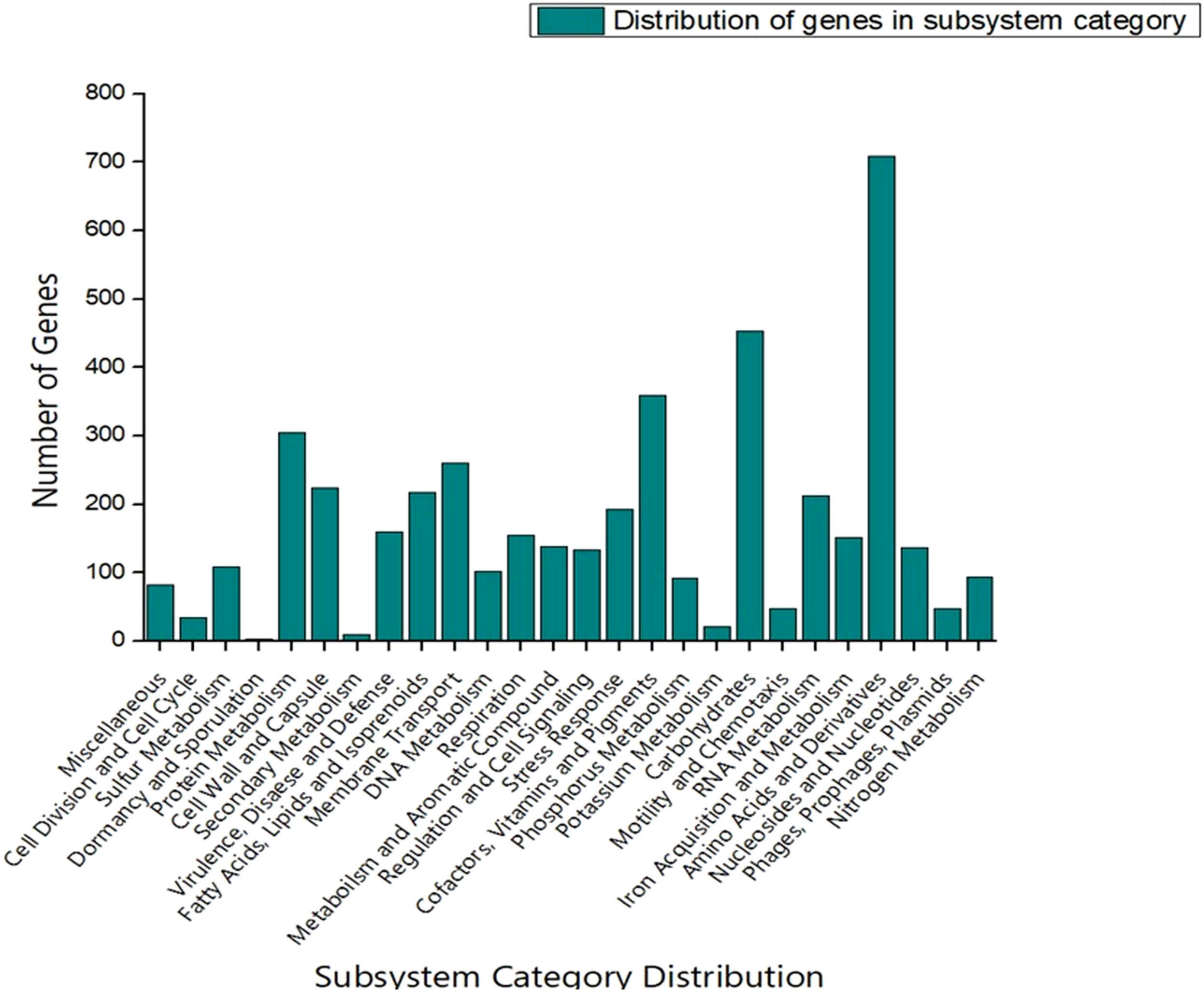
RAST-based comparative analysis of the distribution of genes which showed that S-8 possess a higher number of genes involved in amino acid synthesis, carbohydrate synthesis, cofactors, vitamin & pigments, and protein metabolism etc.

**Figure 4:**
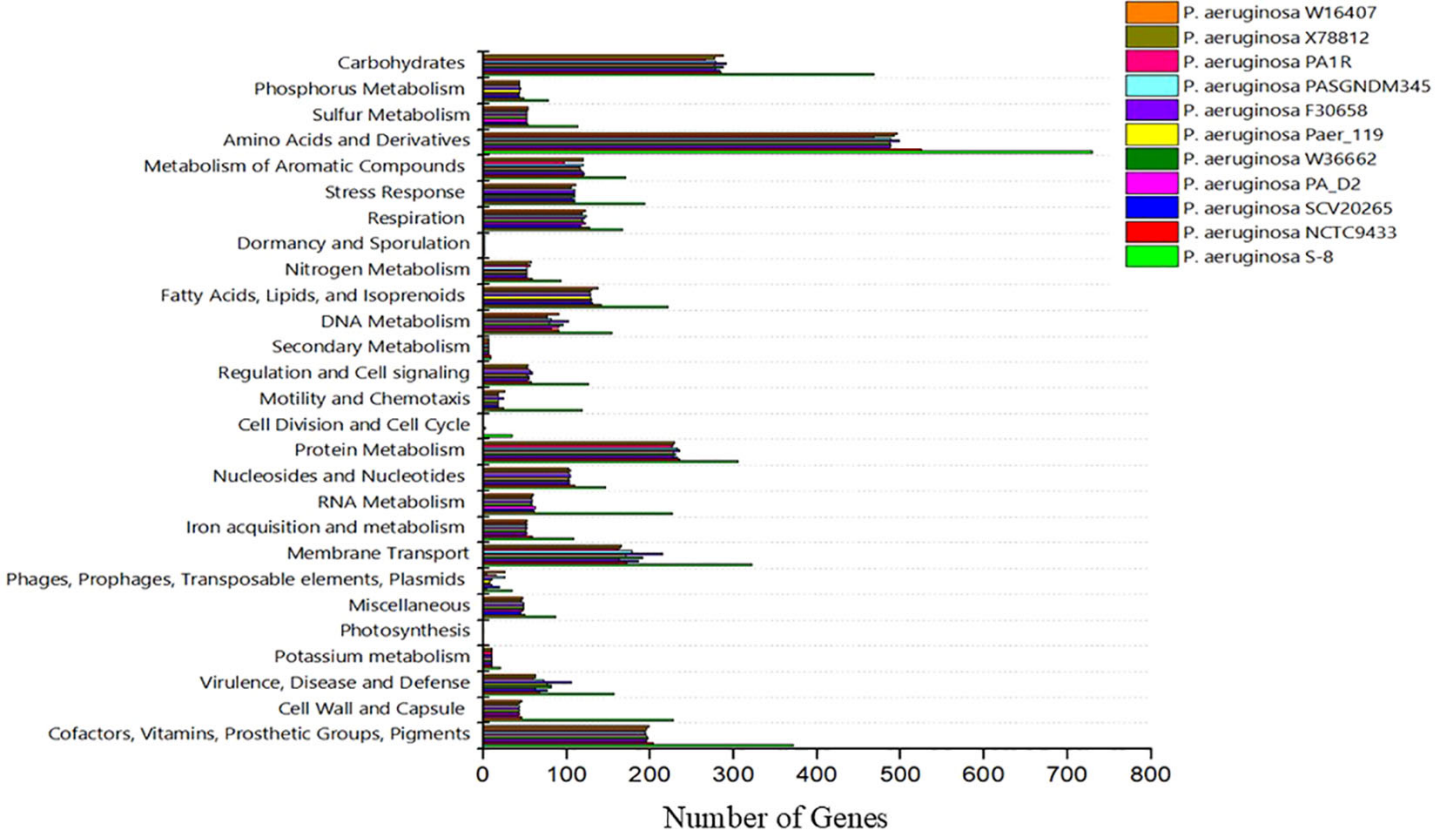
A compassion of gene distribution in the *P. aeruginosa* S-8 genome and its closest relatives genomes including *P. aeruginosa* NCTC9433, *P. aeruginosa* SCV20265, *P. aeruginosa* PA_D2, *P. aeruginosa* W36662, *P. aeruginosa* Paer4_119, *P. aeruginosa* F30658, *P. aeruginosa* PASGNDM345, *P. aeruginosa* PA1R, *P. aeruginosa* X78812, and *P. aeruginosa* W16407.

**Figure 5:**
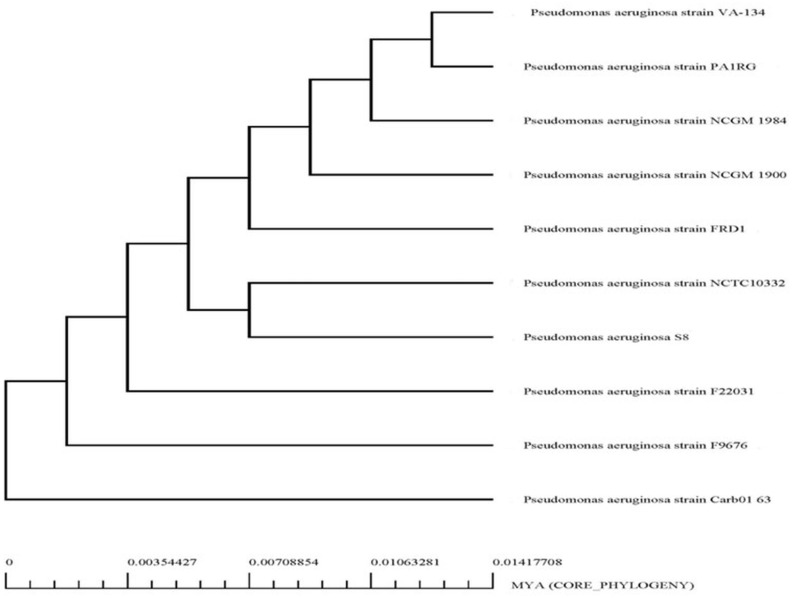
Phylogenetic analysis of *P. aeruginosa* S-8 based on WGS (whole genome sequences). All the closest related reference sequences were represented to PATRIC for phylogenetic analysis. Following MUSCLE alignment, RaxML was used to visualize the matrix.

The genome sequence of S-8 was further annotated by RAST, which showed that the top three subsystem features of S-8 are amino acids derivatives (729 genes), carbohydrates (467 genes), followed by cofactors/vitamins (371 genes) (**Figure 3**). The other subsystem includes the membrane transporter (322 genes), protein metabolism (305 genes), cell wall & capsule (228 genes), RNA metabolism (227 genes), and genes related to fatty acids metabolism (221). The gene annotation was performed with the closest relatives of S-8 including *P. aeruginosa* NCTC9433, *P. aeruginosa* SCV20265, *P. aeruginosa* PA_D2, *P. aeruginosa* W36662, *P. aeruginosa* Paer4_119, *P. aeruginosa* F30658, *P. aeruginosa* PASGNDM345, *P. aeruginosa* PA1R, *P. aeruginosa* X78812, and *P. aeruginosa* W16407 (**Figure 4**; **Supplementary Table 1**). The phylogenomic relationships of S-8 and other *P. agglomerans* strains was established based on the core genome analysis, which showed the closeness of S-8 to *P. aeruginosa* FRD1 and *P. aeruginosa* NCTC 10332 (**Figure 5**).

### Gene ontology

**Figure 6:**
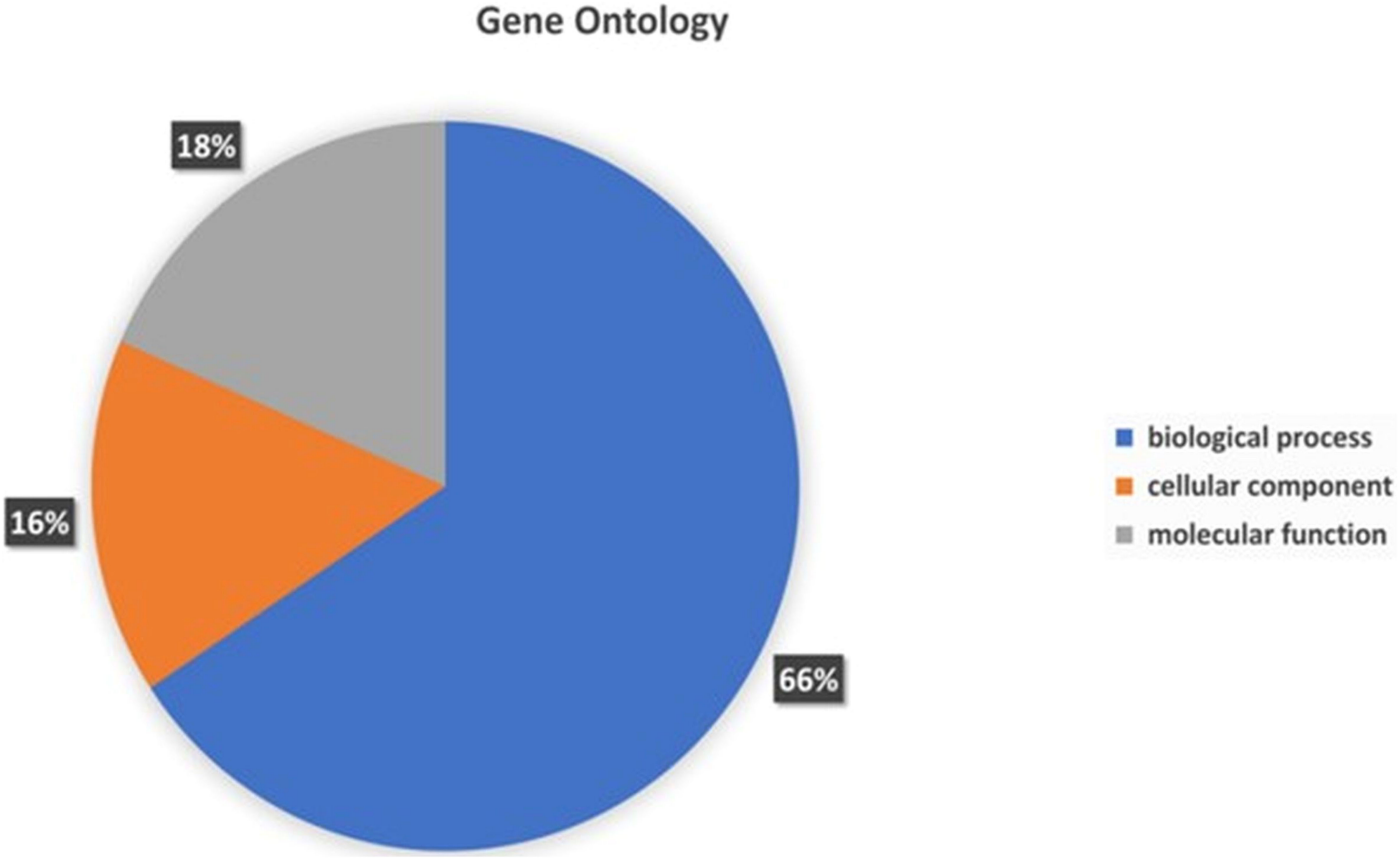
Gene Ontology functional annotations of the *P. aeruginosa* S-8 genome into 3 functional groups; biological processes, cellular component and molecular functions.

To assign the functionality of the classified proteins, gene ontology was investigated. A sum total of 66% of genes were related to biological processes, 18% to molecular functions, and 16% to cellular components (**Figure 6**). In the molecular functions, 23.95% genes were associated with different molecular functions, 15.19% related to catalytic activity, 6.40% to binding processes, 4.96% transferase activity and 3.92% to hydrolase activity (**Supplementary File S1**). In the cellular component, 24.50% of genes were related different cellular component, 23.22% to cellular anatomical entity, 12.23% to intracellular anatomical structure, and 11.67% to cytoplasm (**Supplementary File S1**). In the biological processes, the highest number of genes (8.06%) was observed for different biological processes. The other genes associated with different biological functionality have been summarized in **Supplementary File S1**.

**Figure 7:**
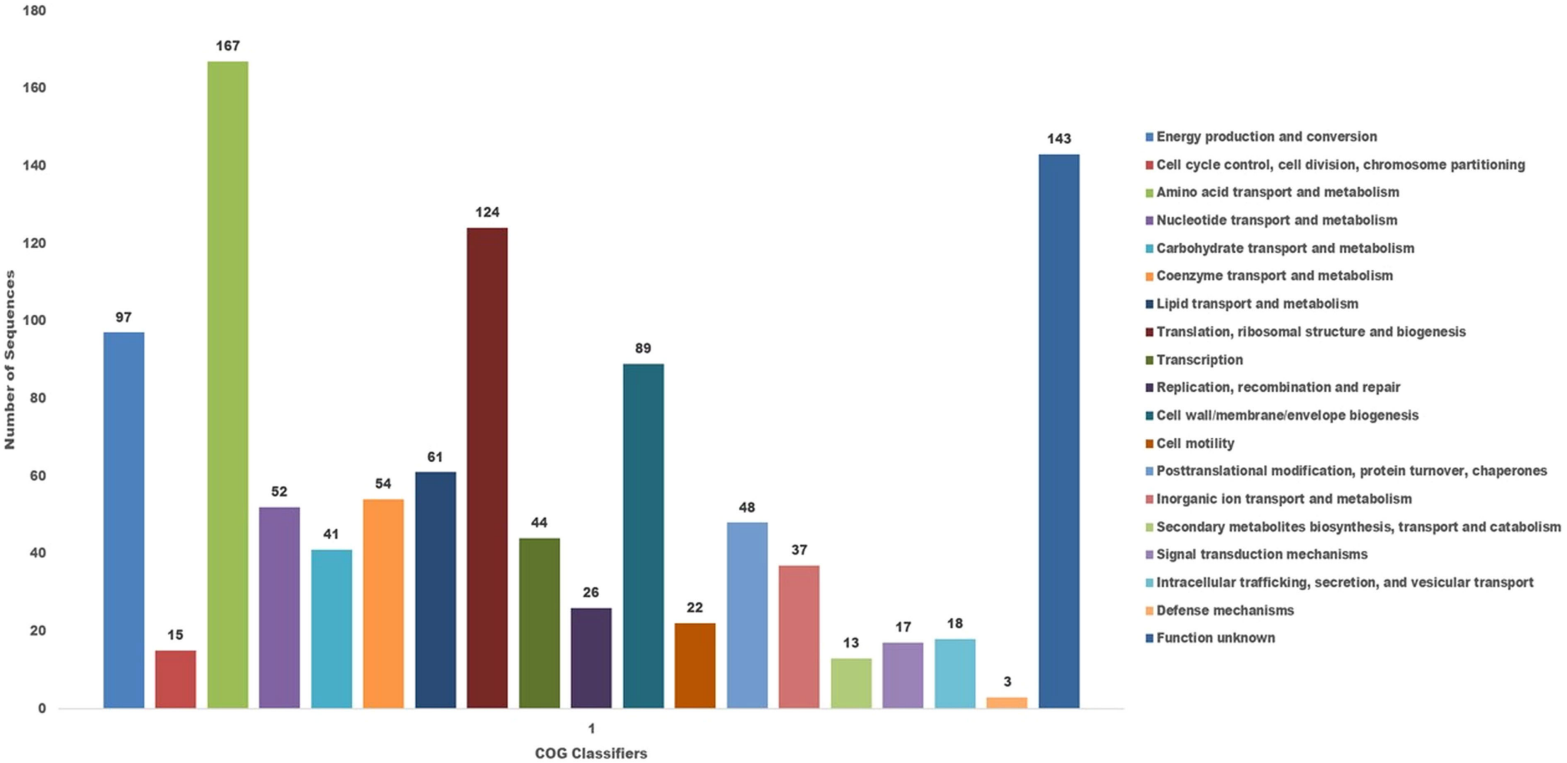
The cluster of orthologus analysis in *P. aeruginosa* S-8 using the COGs database based on orthologous groups. The identified genes in S-8 were divided into several functional subcategories based on the COG annotation (http://www.ncbi.nlm.nih.gov/COG/).

**Figure 8:**
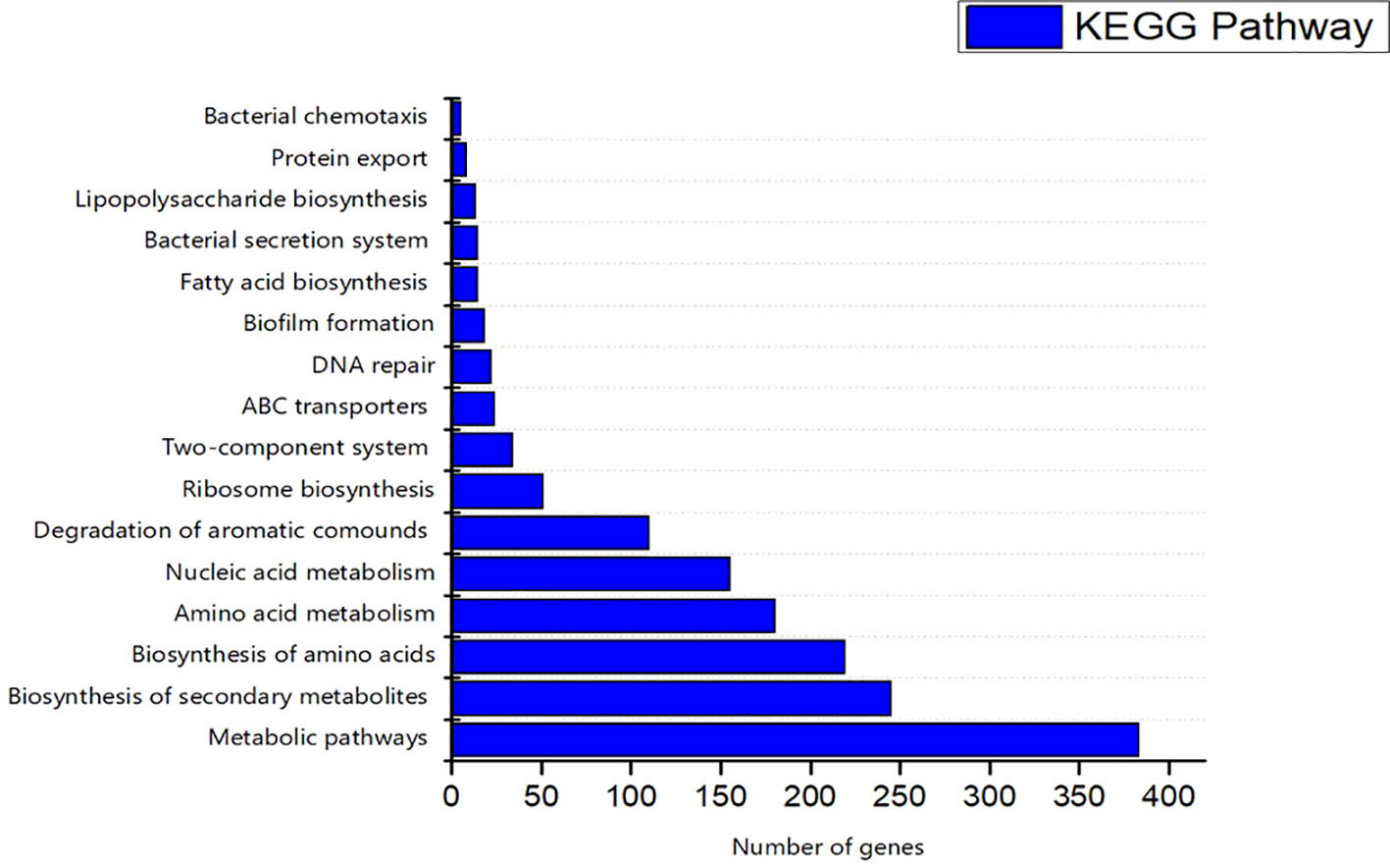
Kyoto Encyclopedia of Genes and Genomes (KEGG) were utilized for the retrieval of top ten (10) metabolic pathways of S-8 genes. KAAS server at KEGG database used for genes functional annotation by BLAST comparison with the manually curated database of KEGG- GENES.

The COG database was used for the functional classification of predicted genes, whose distribution within the COG categories is provided in **Figure 7**. The COG prediction showed a higher number of genes (167), involved in amino acid transport and metabolism, followed by ribosomal structure and biogenesis (124), energy production (97), lipid transport and metabolism (61), coenzyme transport and metabolism (54) and nucleotide metabolism and metabolism (52). A total of 143 genes were identified with unknown functions. KEGG analysis identified the genes belonging to the various metabolic pathways (**Figure 8**). The highest number 383 was recorded for different metabolic pathways, 250 to the biosynthesis of secondary metabolites, 220 to the biosynthesis of amino acids, 170 to amino acid metabolism, 150 to nucleic acid metabolism, 110 to degradation of aromatic compounds, and 50 in the ribosome biosynthesis.

### Average nucleotide identity

To re-evaluate the phylogenetic relationship of S-8 within the *Pseudomonas* genus, ANI percentage of the S-8 genome was calculated with respect to other sequenced *P. aeruginosa* strains (**Supplementary Table 1**). We observed that our genome shows maximum genetic similarity with CP008869.2 (*P. aeruginosa* strain W16407), NC_023149.1 (*P. aeruginosa* SCV20265), CP008872.2 (*P. aeruginosa* strain X78812), and CP013113.1 (*P. aeruginosa* strain PAER4_119) with more than 99.2% similarity (**Supplementary Figure 3**). Similarly, the fastANI also showed the similar trend observed in our primary clustering dendrogram (**Supplementary Figure 4**).

### AMR and VFDB analysis

**Figure 9:**
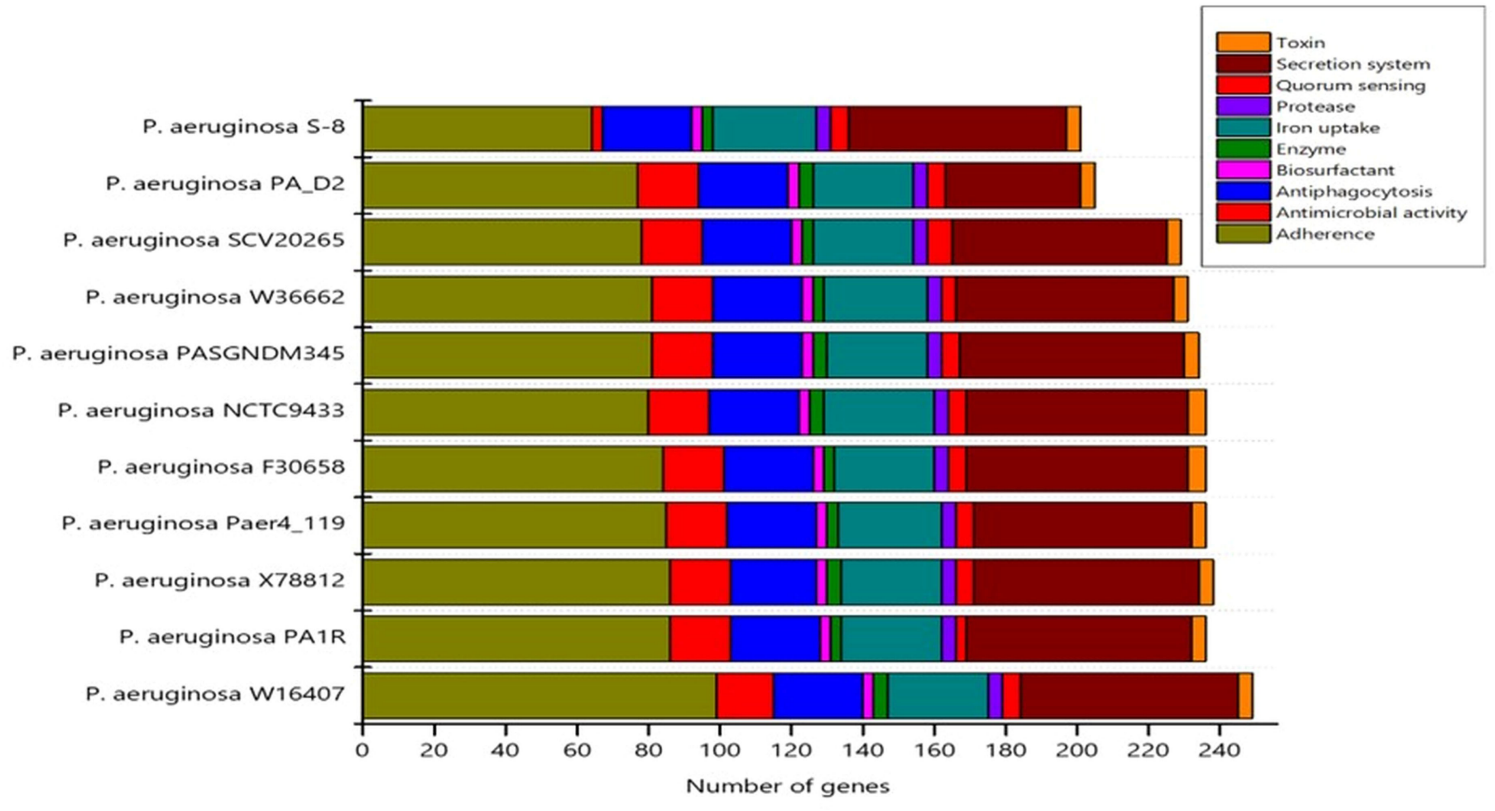
The distribution of virulence genes in the selected genomes was determined by virulence factor data base (VFDB) search tool.

CARD analysis identified the different classes of AMRs which have been summarized in **Supplementary File S2**. A number of resistance genes harbored within the genomes of the S-8 strain were identified and gene families associated to the resistance-nodulation-cell division (RND) antibiotic efflux pump, major facilitator superfamily (MFS) antibiotic efflux pump, beta-lactamase, ATP-binding cassette (ABC) antibiotic efflux pump, multidrug and toxic compound extrusion (MATE) transporter, and pmr phosphoethanolamine transferase were identified, which contributes to antibiotic resistance (**Supplementary File S2**). VFDB analysis identified the genes related to adherence, type IV pili and twitching motility genes, iron uptake related system related to achromobactin, pyoverdine, yersiniabactin biosynthesis, quorum sensing, and GacS/GacA two-component system. Enzymes related to hemolytic phospholipase, biosurfactant, and protease production were noted in S-8 genome. Moreover, genes related to Type VI secretion system (T6SS) like *clpV, hcp, icmF*, and *vgrG* were also annotated (**Supplementary File S3**). A comparison of virulence genes in the selected *Pseudomonas* genome was performed which showed that S-8 has the minimum virulence genes in all categories including adherence, antimicrobial activity, anti-phagocytosis, secretion system, protease activity, and toxin production, etc (**Figure 9**).

### BGCs analysis

**Figure 10:**
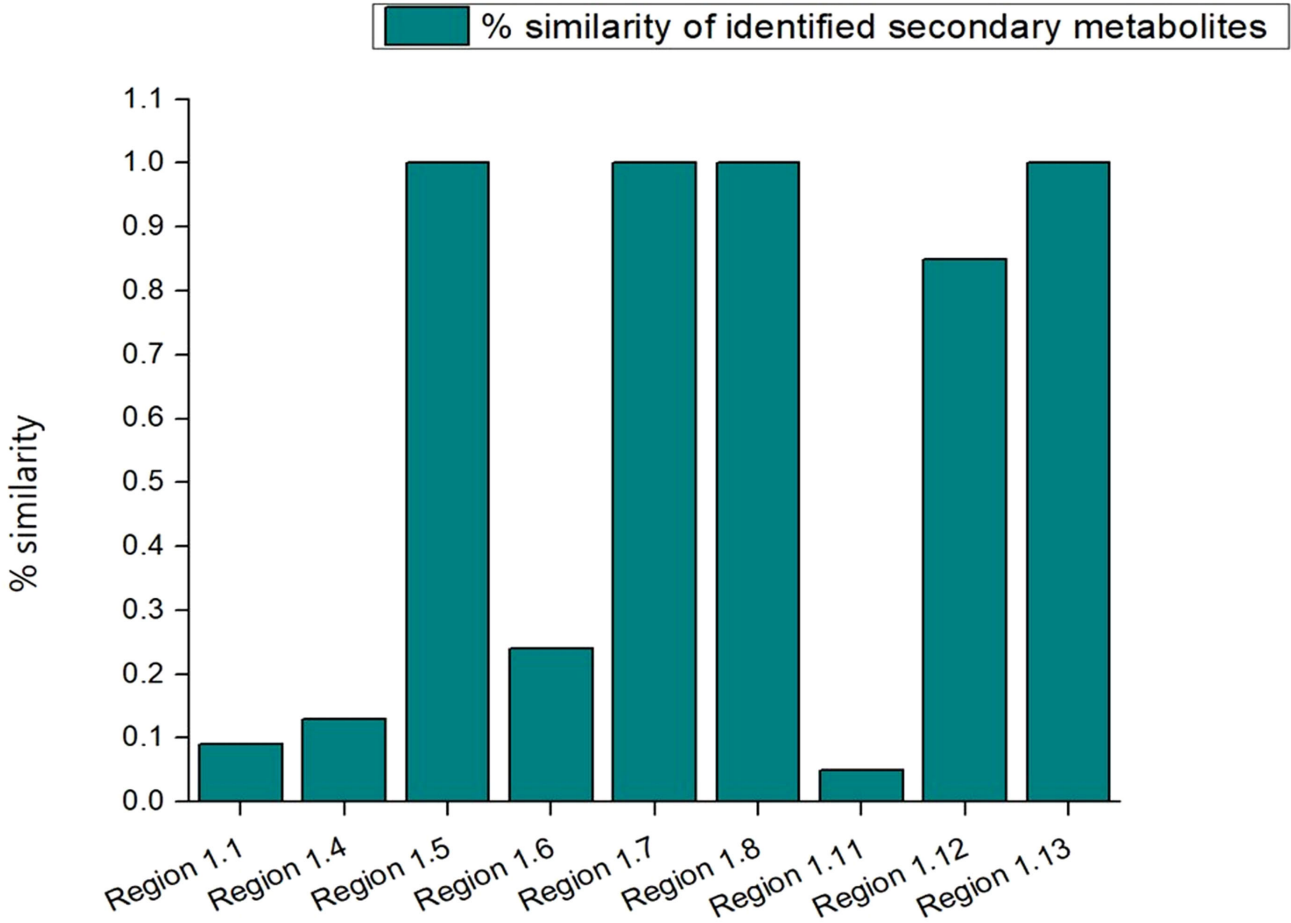
A total of 13 BGCs detected by antiSMASH in *P. aeruginosa* S-8 genome. The identified BGCs were belonging to different category like NRPS- (non-ribosomal peptides synthase) like betalactone, thiopeptide, NRP-metallophore, and PKs (polyketides synthase).

In order to mine the secondary metabolite pathways, the various BGCs in the strain S-8 genome were annotated using the antiSMASH database, and revealed thirteen different secondary metabolites regions in the genome (**Figure 10**). In terms of biosynthetic paradigms, BGCs were comprised of non-ribosomal polypeptides (NRPS) like betalactone cluster, thiopeptide, hserlactone, redox-cofactor, and NRP- metallophore. The four NRPS clusters found in S-8 showed 100% similarity, to 2-amino-4-methoxy-*trans*-3-butenoic acid, pyoluterin, azetidomonamide, and pseudopaline, respectively (**Table 2**).

**Table 2:** Annotated BGCs in the *P. aeruginosa* S-8 genome.

Region	Type	From	To	Most similar known cluster	Peptides	Similarity
Region 1.1	NRPS-like betalactone	1,310,054	1,350,182	Pyoverdine SMX-1	NRP	9%
Region 1.2	hserlactone	1,556,574	1,576,859			
Region 1.3	thiopeptide	2,083,578	2,116,581			
Region 1.4	redox-cofactor	2,160,030	2,182,174	Lankacidin C	NRP+Poly ketide	13%
Region 1.5	NRPS	2,506,061	2,556,547	L-2-amino-4-methoxy-trans-3- butanoic acid	NRP	100%
Region 1.6	NRP-metallophore, NRPS	2,606,092	2,723,061	Pf-5 pyoverdine	NRP	24%
Region 1.7	TIPKS. T3PKS	2,906,603	2,965,103	Pyoluteorin	Polyketide	100%
Region 1.8	NRPS	3,784,399	3,831,236	Azetidomonamide A/B	NRP	100%
Region 1.9	NAGGN	3,936,792	3,951,552			
Region 1.10	Hserlactone (Homoserine- lactone)	3,954,341	3,974,946			
Region 1.11	NRPS-like	4,642,583	4,684,572	MA026	NRP	5%
Region 1.12	NRP-metallophore,NRPS	4,819,411	4,673,917	Pyochelin	NRP	85%
Region 1.13	Opine-like-metallophore	5,587,818	5,609,907	pseudopaline	Other	100%

### CAZymes analysis

**Figure 11:**
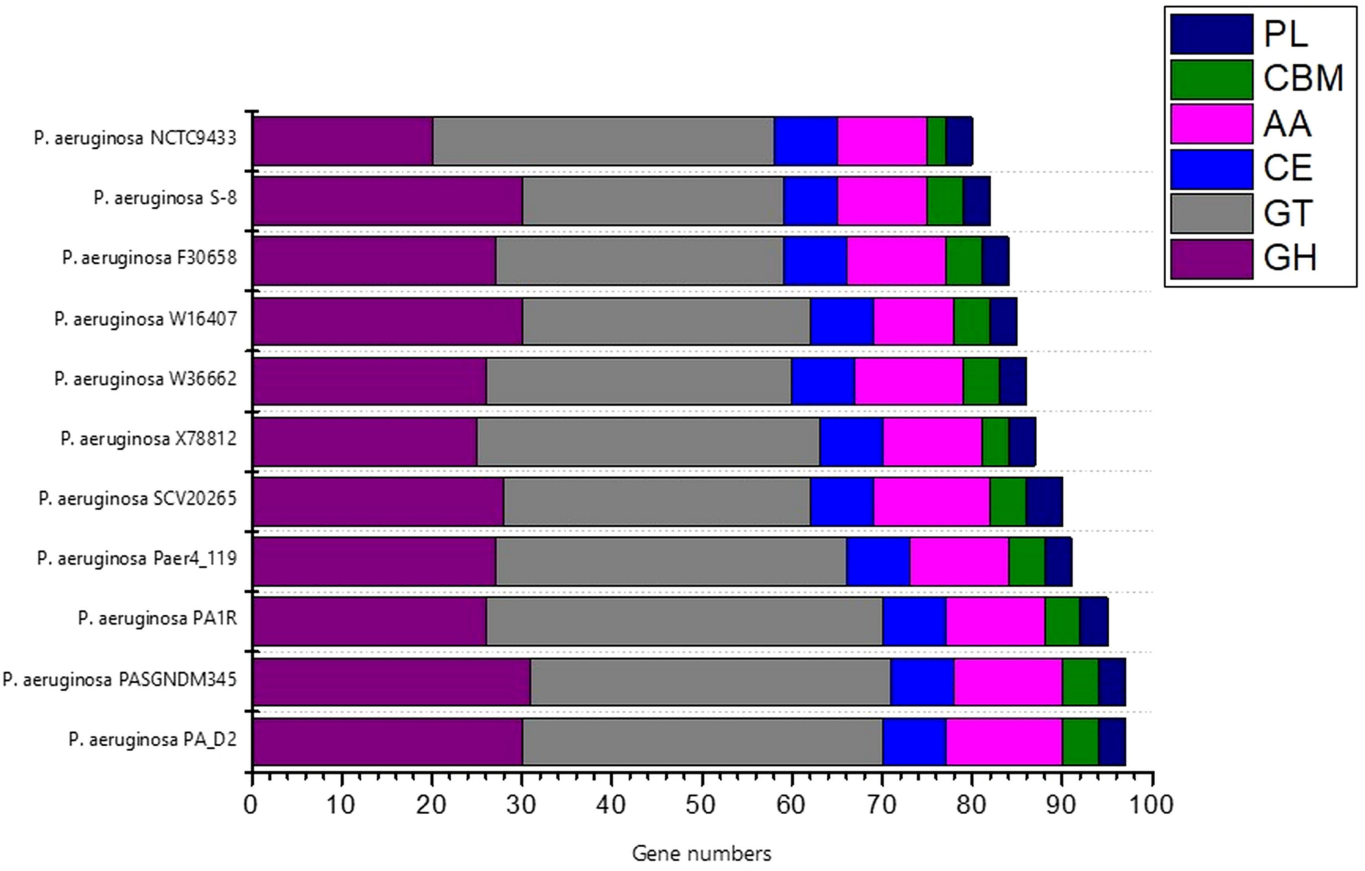
The distribution of various CAZymes like carbohydrate-binding modules (CBMs), glycoside hydrolases (GHs), glycosyl transferases (GTs), polysaccharide lyases (PLs), carbohydrate esterases (CEs), and auxiliary activities (AAs) in various *P. aeruginosa* species.

To investigate the industrial-relevant enzymes involved in the breakdown of complex carbohydrates, the S-8 genome was analyzed by the dbCAN2 server. As a result, 82 CAZymes genes were identified in the S-8 genome, which was classified into glycoside hydrolases (GHs), glycosyltransferases (GTs), carbohydrate-binding molecules (CBMs), carbohydrate esterases (CEs), and auxillary activities (AAs). Of these, the most abundant CAZymes were GHs and GTs with 30 and 29 genes, respectively, followed by AAs (10 genes), CE (6 genes), CBMs (4 genes), and PLs (3 genes) (**Figure 11**). The different groups of CAZymes were also compared to other ten genomes of *P. aeruginosa* strains (**Supplementary Table 1**) and a comparison of different CAZymes has been demonstrated in **Figure 11**. Among the compared genome, a higher number of CAZymes was observed for *P. aeruginosa* PA_D2 and *P. aeruginosa* PASGNDM345, followed by *P. aeruginosa* PA1R. The lowest diversity of CAZymes was noted for *P. aeruginosa* NCTC9433.

### Comparative genome analysis

**Figure 12:**
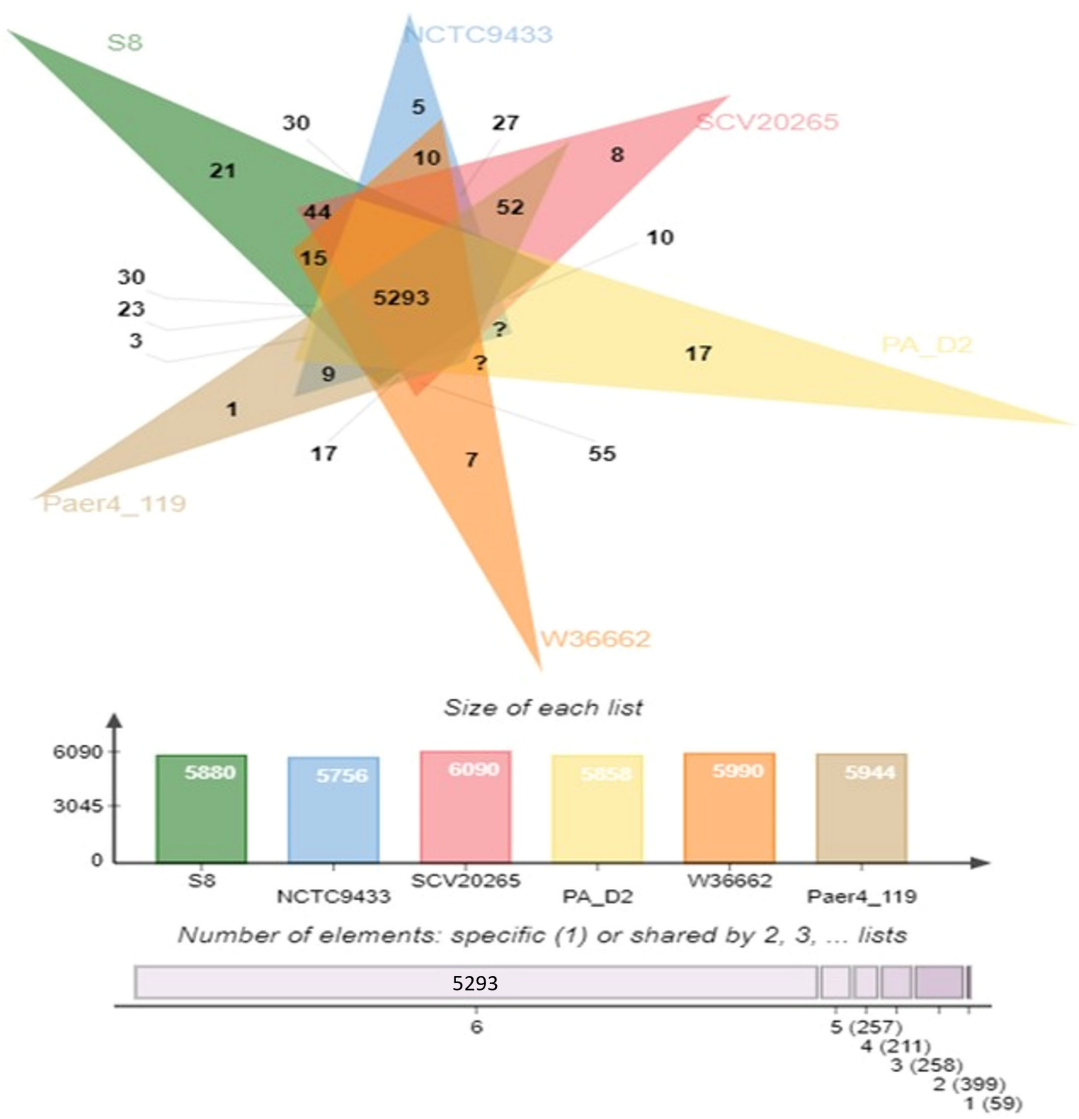
Venn diagram generated by Orthovenn2 represents the distribution of shared and unique gene clusters among selected *Pseudomonas* genomes. Compared to six *Pseudomonas* strains, the S-8 strain had twenty-one unique genes, however, a total of 5293 gene were shared among all six strains.

**Figure 13:**
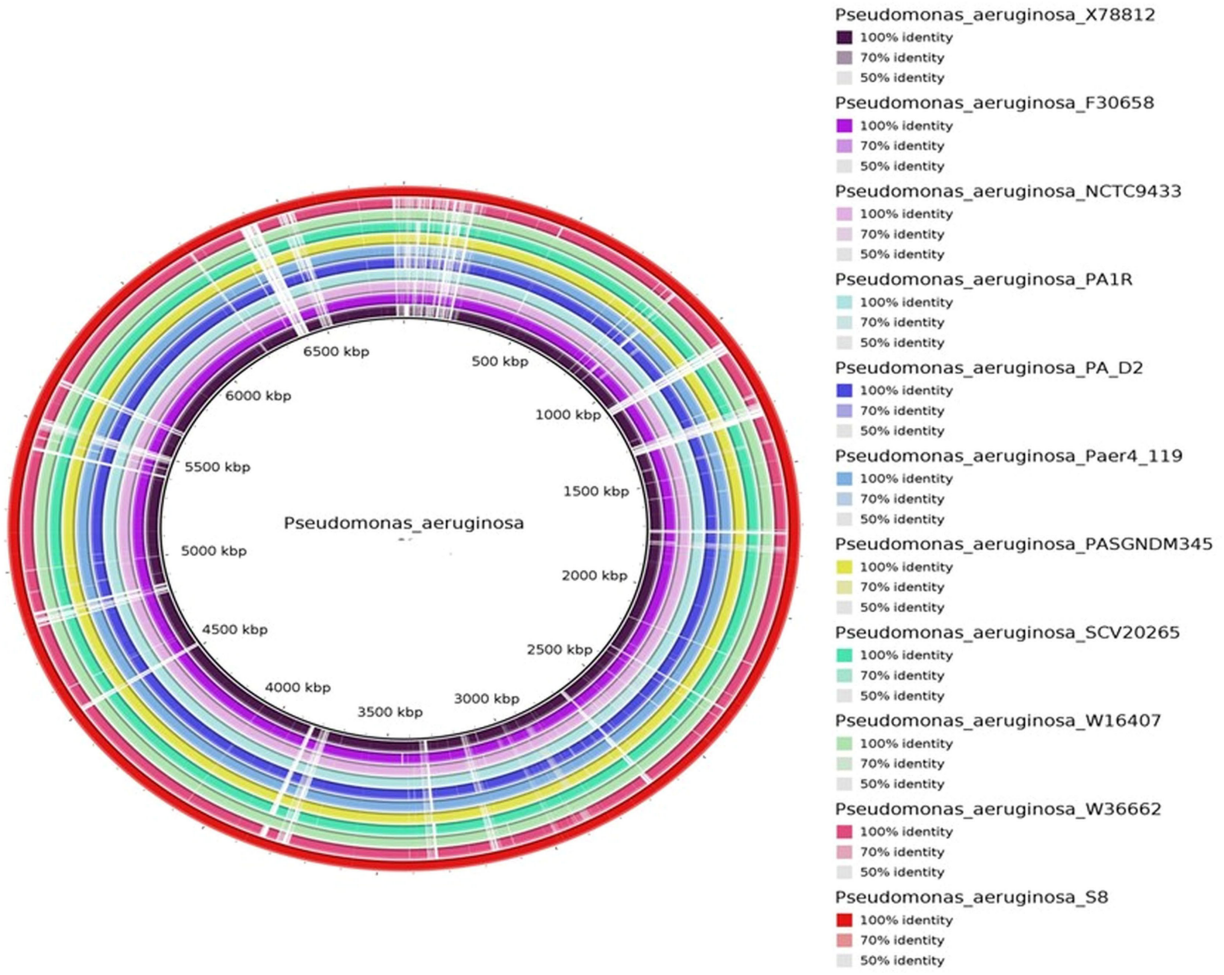
The circular genome comparison of assembled genome of S-8 was performed against the reference genome of *P. aeruginosa* X78812, *P. aeruginosa* F30658*, P. aeruginosa* NCTC9433, *P. aeruginosa* PA1R, *P. aeruginosa* PA_D2, *P. aeruginosa* Paer4_119*, P. aeruginosa* PASGNDM 345, *P. aeruginosa* SCV20265, *P. aeruginosa* W16407, and *P. aeruginosa* W3662 by using Blast Ring Image Generator (BRIG) (v 0.95) Tool.

The protein coding gene comparison was performed between S-8 and the other closely related strains. The Venn diagram and the bar plot (**Figure 12**) showed that the numbers of core ortholog clusters shared by all the six species were 5,293, that suggests their conservation in the lineage after speciation events. The cumulative number of ortholog clusters shared between any two genomes, including the OS-1 was 30. A total of 59 gene clusters were unique to only a single genome. These clusters are probably gene clusters within multiple genes or in-paralog clusters which suggest that a lineage-specific gene expansion has occurred in these gene families. Additionally, the bar plot below the Venn diagram showed that the number of ortholog clusters for each species varied; *P. aeruginosa* S-8 (5,880), *P. aeruginosa* NCTC9433 (5,756), *P. aeruginosa* SCV20265 (6,090), *P. aeruginosa* PA_D2 (5,858), *P. aeruginosa* W36662 (5,990), and *P. aeruginosa* Paer4_119 (5,944). The formation of gene clusters was the first pattern, the second pattern shows the cluster counts and the third pattern represented in the form of stacker bar shows the total protein counts (**Supplementary Figure 5A**). The pairwise heatmap was performed for S-8 and other strains to highlight the overlapping number of gene clusters (**Supplementary Figure 5B**). A red color gradient showing the highest overlapping gene cluster thresholds was noted between *P. aeruginosa* S-8 and *P. aeruginosa* W16407 (**Supplementary Figure 5B**). A circular comparison performed by BRIG revealed the overall genome of *P. aeruginosa* S-8 has a high degree of sequence similarities (>99%) with other compared genomes (**Figure 13**).

### Pan-genome analysis

**Figure 14:**
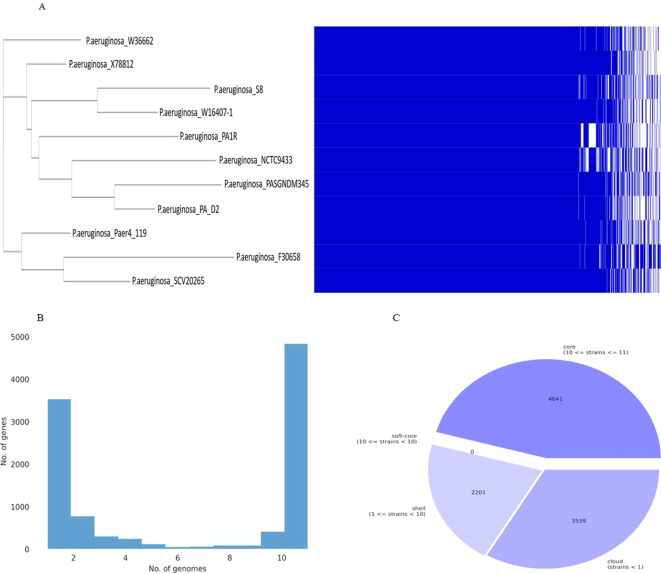
Graph of the pan-genome distribution of *P. aeruginosa* S-8 and other closely related genomes; **(A)** Gene distribution of *P. aeruginosa* strains based on the gene presence–absence matrix generated from Roary. A purple box, a green box, and an orange box to represent the core gene, accessory genes and specific genes, respectively; **(B)** The gene frequency plot within a whole genome set, demonstrating the distribution of genes per genome **(C)** Pie-chart representation of the gene distribution in the pan-genome with respect to presence of genes in proportion of strains out of ten strains selected.

The pan-genome and core-genome analysis was performed using the 10 genomes of the closest strains and the constructed tree showed two clusters based on presence/absence gene profiles (**Figure 14**). The resulting core genome SNPs-based phylogeny could be a good alternative for a better resolution than ANI-based analysis. For all 11 strains including *P. aeruginosa* S-8, the pan-genome contains 10,581 genes, of which the core genome represents 4,841 genes (approximately 40%), whereas the shell and cloud genome represents 2,201 (approximately 21%), and 3,539 genes (approximately 34%), respectively (**Figures 14A-C**). The core genome represented the largest part of the gene pools, this richness confirms the diversity and multiplicity of diverse traits. On average each strain contained 310 unique genes which correspond to approximately 3.0% of each genome. Box-plot analysis showed that among the selected strains, S-8 showed the less conserved genes (**Supplementary Figure 6A**), and a high number of unique genes (**Supplementary Figure 6B**).

### Horizontal gene transfer analysis

**Figure 15:**
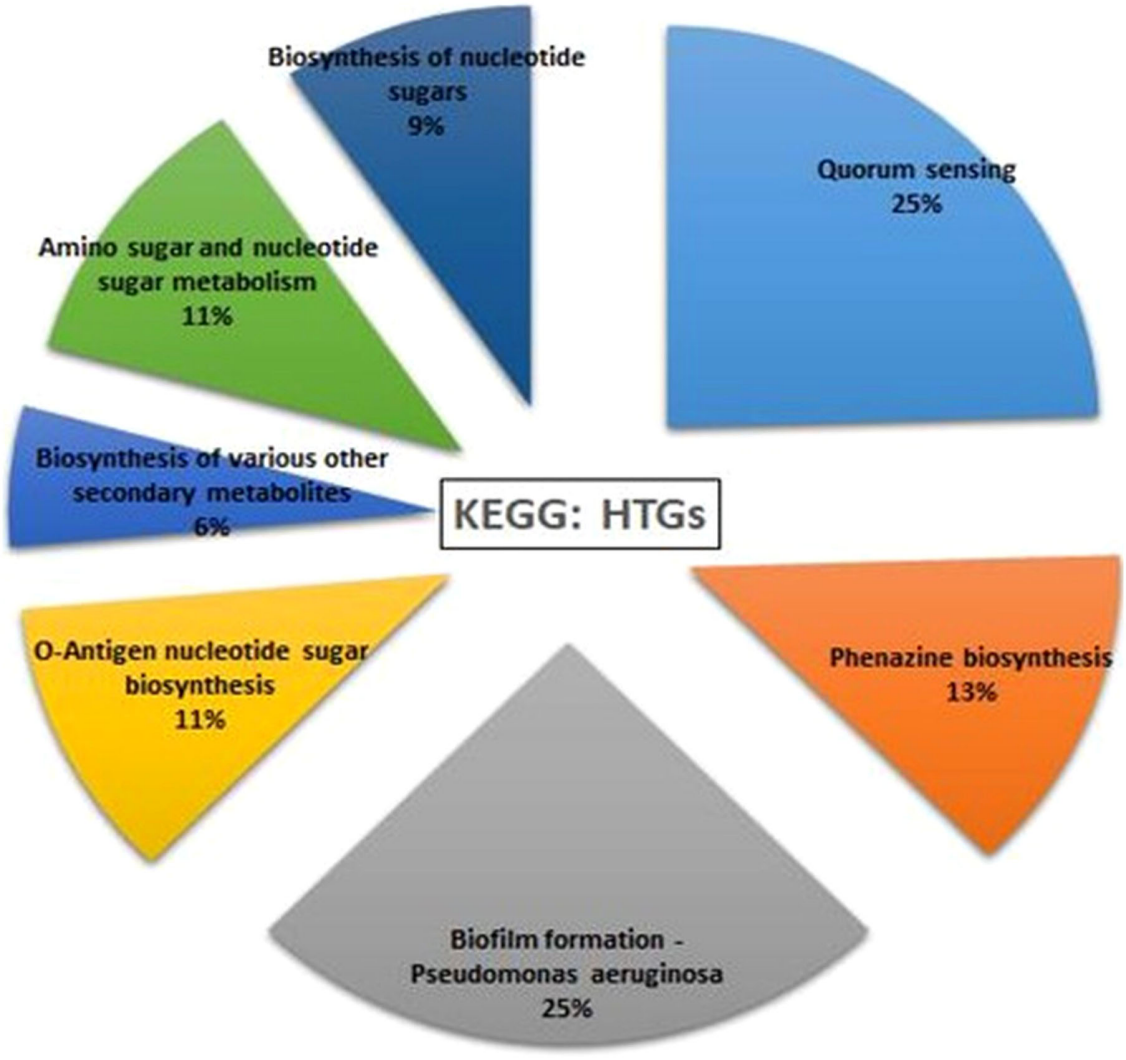
Horizontal gene transfer event was analyzed by HGTector2 which showed a total of 911 HGTs form our S8 genome. After identifying the horizontal gene transfers (HGTs) within the genome of interest, we conducted a KEGG pathway analysis specifically for these HGTs.

**Figure 16:**
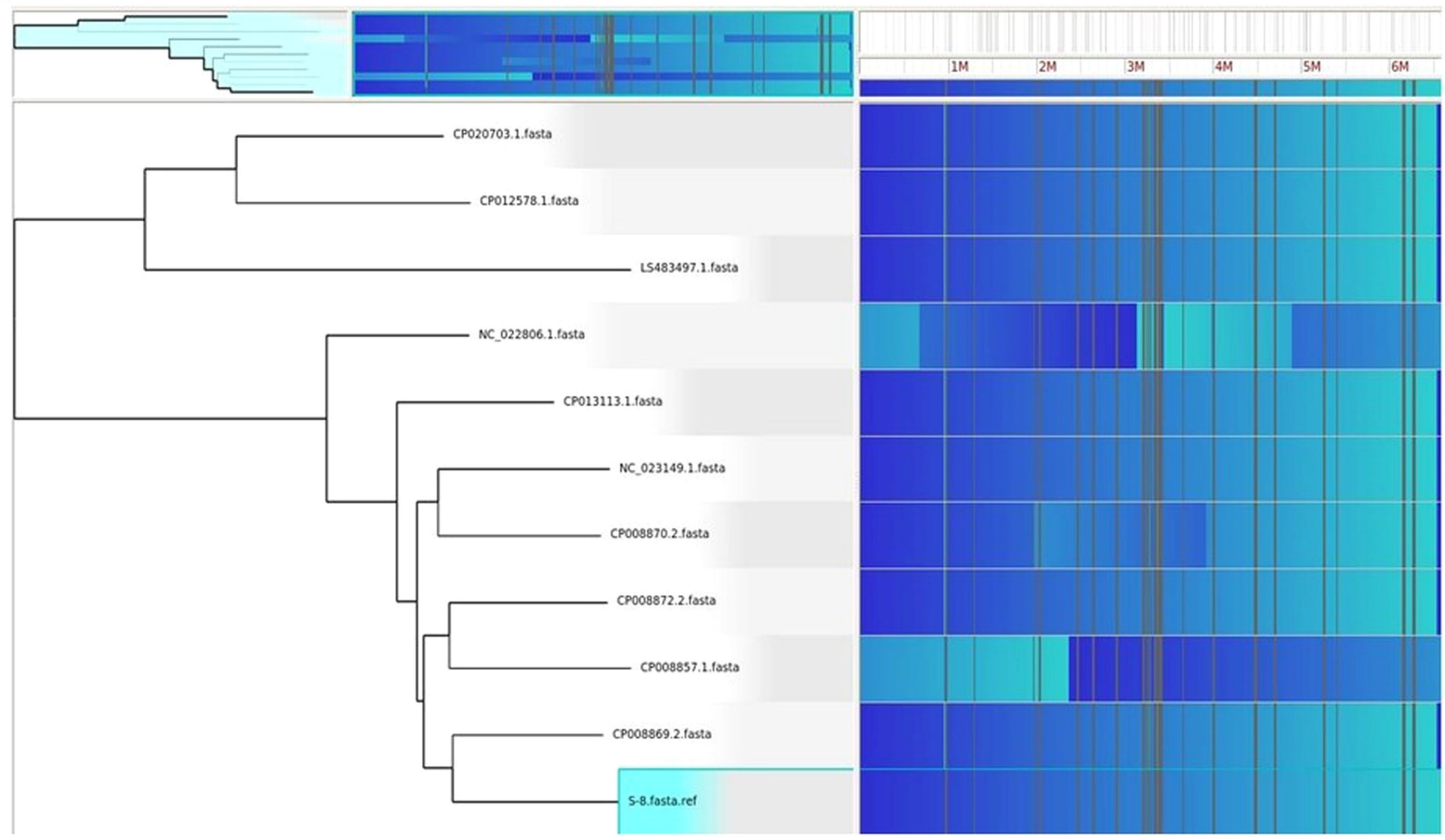
The Gingr generated tree based on parsnp analysis. The tree provides a visual representation of the genetic clustering and divergence patterns among the analyzed strains of *Pseudomonas.* The proximity of branches indicates a more recent common ancestry, while greater distances reflect greater genetic divergence.

HGTector2 analysis resulted in 911 HGTs form our S8 genome. Considering all genomes, we found a total of 10,547 genes. After identifying the horizontal gene transfers (HGTs) within the genome of interest, which in this case is S8, we conducted a KEGG pathway analysis specifically for these HGTs. The results showed that higher number (25%) was observed for the Quorum sensing and biofilm formation followed by phenazine biosynthesis (11%), amino acids and nucleotide metabolism (11%), and nucleotide sugar biosynthesis (11%) (**Figure 15**). The Parsnp based SNP tree showed a visual representation of the genetic clustering and divergence patterns among the analyzed strains of *Pseudomonas*. It represents a genome alignment approach to determine the precision of mapping, but with a reduced sensitivity. Analysis (**Figure 16**) showed that there was less variation between S-8 and *P. aeruginosa* W16407, whereas a high variation was observed between S-8 and *P. aeruginosa* PA1R.

## Discussion

Utilizing the sequencing data of newly isolated strains, it is possible to make *in silico*-based predictions about the gene repository and virulence potential of a newly isolated strain *P. aeruginosa* S-8. This robust method decreases the need for a laborious trial, error-type wet lab experiments, and animal testing. In the present work, we investigated the in-depth genome, pan-genome, and comparative genome analysis of *P. aeruginosa* to assess the genomic differences and similarities between closely related strains. This type of study will provide insights into the adaption process of an environmental isolate to a particular environmental habitat. Moreover, the environmental isolates due to adaption to different habitats show more closeness among themselves than the clinical isolates (Sánchez et al., 2014). The genomes of several *Pseudomonas*-type strains have been deciphered, which has contributed to an improved understanding of the evolution and diversity of the *Pseudomonas* genus (Silby et al., 2011).

In particular, more *P. aeruginosa* isolates are being sequenced and showed the presence of various pathogenicity and virulence features (Klockgether et al., 2011; Marcelletti et al., 2011). Among the *Pseudomonas* genus, *P. syringae* comprise plant pathogens secreting effector’s proteins into plant cells by the type III secretion system (Lindeberg et al., 2012), whereas, several *P. fluorescens* are known for their biocontrol properties (Haas and Defago, 2005). In contrast, *P. putida* strains are mostly non-pathogenic and show robust metabolic capacities under stress conditions (Rojas et al., 2001). Flagella are used by bacteria for motility and many genes for flagella formation were annotated in the S-8 strain. The presence of flagella improves bacterial motility and consequently pathogenicity, and also provides the bacterium resistance to surfactant protein A (SP-A) (Zhang et al., 2007), a potent lung innate immune protein that kills microbial pathogens through opsonization (Tan et al., 2014a). Additionally, specific pili like type IV pili enhance the motility and antimicrobial resistance in *P. aeruginosa* (Tan et al., 2014b), and have been reported in severe cases of pneumonia, bacteremia, and increased mortality (Tang et al., 1995).

The KEGG analysis revealed that strain S-8 harbored the genes for the gluconeogenesis pathway, pentose phosphate pathway, purine and pyrimidine synthesis, fatty acid and peptidoglycan synthesis pathway. In terms of nitrogen metabolism, S-8 could take up ammonia using the ammonium family transporters. Besides sulfur metabolism, strain S-8 harbored the gene set for sulfate, phosphate, alaknesulfate and lipopolysaccharide, which enable S-8 to obtain and utilize nutrients such as nitrogen, carbon, phosphorus and sulfur from the environment to facilitate its survival in different environments. The genome analysis showed that S-8 could synthesize various amino acids including alanine, glycine, glutamate, serine, threonine, cysteine, valine, glutamine, proline, arginine, and phenylalanine. Moreover, genes for vitamin metabolism, peroxidase, superoxide dismutase, lipopolysaccharide biosynthesis, β-lactam resistance, and cationic antimicrobial peptide were also noted, which could enable the *Pseudomonas* strain S-8 to adapt to a complex and changeable environment.

Genome annotation identified the various metal resistance genes in S-8 genome. Genes coding for copper-translocating P-type ATPases (*opA, copB*, *cusR, cusS*, *cueR*) were identified which are a large family of transmembrane transporters and their role in ion homeostasis and tolerance of heavy-metal ions is well established. A previous study showed that P-type ATPases pumped out the metal ions from the cytoplasm to the periplasm. The *cusRS* is a two-component signal transduction system that activates the expression of *cus*CFBA operon and is involved in copper detoxification (Gilles-Gonzalez and Gonzalez, 2005). Copper-translocating P-type ATPases acts as an efflux pump in order to pump out excess Cu^2+^ from the cell (Ladomersky and Petris, 2015). Genes involved in lead resistance *zntA* and *cadC*, arsenic resistance *arsC, arsR*, genes involved in cobalt resistance *czcD*, and chromium resistance *chrA* were detected (**Supplementary Table 2**). Nickel-responsive regulator protein *nikR* and copper chaperone protein *copZ* were noted which belong to drug/metabolite transporter superfamily, and are presumed to expel the toxic metabolites out of the cell (Gudhka et al., 2015). *P. aeruginosa* S-8 was motile and we identified chemotaxis regulator proteins encoded by *CheA, CheB, CheB1, CheR, CheV, CheY, CheW*, and *CheZ* gene. Additionally, chemotaxis-associated proteins encoded by *McpC, McpH, McpP, McpQ, PctA, PctB* and *CtpH* genes along with flagellar proteins (*FlhABF, FliCDEFGOP, MotAB*) were also identified. Genes involved in the regulation of Fe uptake *e.g. Fur* and *PchhR* were detected in the S-8 genome (**Supplementary Table 3**).

S-8 genome also showed the presence of osmotic stress genes e.g. *mdoB* (Phosphoglycerol transferase I), *mdoH* (Glucans biosynthesis glucosyltransferase H), *mdoG* (Glucans biosynthesis protein G precursor), and *mdoD (*Glucans biosynthesis protein D precursor). Production of cold-shock and heat-shock proteins by microorganisms can help their survival in harsh environments and facilitate environmental adaption. The S-8 genome carries the various heat-shock and the cold-shock protein (**Supplementary Table 4**). Moreover, the S-8 genome encodes numerous proteins to protect the cell from oxidative stress including five peroxidases, three catalases, four superoxide dismutases, two hydroperoxide reductases and 11 glutathione S-transferases (GST). GST is a detoxification enzyme involved in cell protection against stress-induced reactive oxygen species (Oakley, 2011). Furthermore, many dehydrogenases/oxidoreductases were found in the S-8 genomes which play an important role as an oxidative protection in bacteria in response to heavy metals (Kaur et al., 2006; Williams et al., 2007). S-8 genome also encoded ions scavenging systems, such as thioredoxin (1 gene), thioredoxin reductase (2 genes), ferredoxin (3 genes), and glutaredoxin (2 gene) which act as defense proteins to protect microorganisms from oxidative damage (Gorriti et al., 2014).

AntiSMASH informatics predicted that S-8 genome has many secondary metabolite synthesis gene clusters with gene cluster types such as NRPS, PKS, T3PKs, and was able to predict pyoverdine, lankacidin, pyochelin, and pseudopaline etc. These gene clusters may all be involved in the antimicrobial process. It has been shown in the literature that pyoverdine, as a broad-spectrum antibacterial substance present in many *Pseudomonas* spp., exhibits bactericidal activity mainly by destabilizing the phospholipid membrane of the target pathogen, leading to cell lysis (Liu et al., 2021). The biosynthetic gene clusters of pyochelin has demonstrated other biological activity recently other than being only a chelating compound. This compound can particularly inhibit bacterial pathogens in a study conducted by Adler et al. (2012) and Ong et al. (2016). In this study, we found that S-8 contains a cluster of biosynthetic genes for many of the above-mentioned inhibitory substances based on genome sequencing, indicating that the bacterium not only has broad inhibitory activity against bacteria but also has the potential to inhibit pathogenic fungi.

Secondary metabolites are organic molecules that have diverse and powerful biological functions which facilitate the bacterial strain to adapt to the environment (Abegaz and Kinfe, 2019). The respective genes involved in the synthesis of these secondary metabolites are often clustered into biosynthetic gene clusters (Medema et al., 2015). In S-8 genome, we identified several gene clusters for secondary metabolites production which act as defensive molecules for the organism producing them (James, 2017). Non-ribosomal peptides (NRPs) are synthesized through enzyme-mediated condensation of amino acid residues where > 300 different precursor molecules help in the assemblage of NRPs (Saxena et al., 2020), and fall into the class of secondary metabolites with diverse properties as toxins, siderophores, antibiotics, immune-suppressants, and anticancer agents (Wang et al., 2014; Martínez-Núñez and López, 2016). All these BGCs comprised approximately 14.6% of the genome. Various gene clusters associated to NRPSs toxic for prokaryotes and eukaryotes were identified in S-8 genome (Rojas Murcia et al., 2015). Some of the NRPSs clusters showed similarity to the pyoverdine cluster with less than 100% similarity. Pyoverdine is a common siderophore found within *P*. *aeruginosa* species and could represent a novel drug or vaccine target (Fothergill et al., 2012).

Region 1.5 represented the clusters of gene for the biosynthesis of bicarbonate transport ATP-binding protein CmpD, nitrate import ATP-binding protein NrtD, cysteine/O-acetylserine efflux protein, purine ribonucleoside efflux pump NepI, alpha-ketoglutarate-dependent taurine dioxygenase, gamma-glutamyl putrescine oxidoreductase, and FMNH2-dependent monooxygenase SfnC (**Supplementary Table 6**). The nitrate import ATP-binding protein facilitates the nitrate uptake, nitrite transport, and responsible for energy coupling to the transport system (Watzer et al., 2019). The bicarbonate transport ATP-binding protein is involved in the specific and high affinity binding of nitrate and nitrite, and shows structural similarities to integral membrane subunits of ABC transporters (Poschenrieder et al., 2018).

The seventh cluster (Region 1.7) contained genes for the various transporters belonging to antibiotic efflux pump outer membrane protein (ArpC), multidrug ABC transporter permease (YbhR, YbhS, YbhF), gramicidin dehydrogenase (LgrE), vitamin B12 transporter (BtuB), L-lactate transporter, and helix-turn-helix (HTH)-type transcriptional regulator (MalT, YofA, DmlR) (**Supplementary Table 7**). The antibiotic efflux pump outer membrane protein form trimeric channels that facilitate the export of a variety of substrates in gram-negative bacteria, whereas, ABC transporter complex YbhFSR could be involved in efflux of cefoperazone (Feng et al., 2020). Vitamin B_12_ (or cobalamin) is an enzymatic cofactor essential for bacteria, however, it can be synthesized only by few microorganisms, so most bacteria need to take it up from the environment through the TonB-dependent transport system. Its import to bacterial cells occurs through the outer-membrane receptor called BtuB which forms a *β*-barrel with inner luminal domain and extracellular loops. Vitamin B_12_ binds with high affinity to the extracellular side of the BtuB protein (Pieńko and Trylska, 2021). The HTH family of transcription regulators is involved in the development of antibiotic resistance (Eckstein et al., 2021). Another enzymes, methane and alkanesulfonate monooxygenase belongs to the family of oxidoreductases and catalyzes the chemical reaction with O_2_ as oxidant and incorporation or reduction of oxygen (Liew et al., 2021).

The 8^th^ cluster (Region 1.8) contained various genes for phosphoethanolamine transferase (EptA), phosphate-import ATP-binding protein (PhnC), acyl carrier protein, inner membrane transport protein (YdhP), ABC transporter phosphite binding protein (PhnD1), and transcriptional regulator (SlyA) (**Supplementary Table 8**). The phosphoethanolamine transferase (EptA) is an intramembrane enzyme that modifies the lipid-A portion of lipopolysaccharide (LPS) or lipooligosaccharide (LOS) by the addition of phosphoethanolamine, which resulted in reduction of overall net-negative charge of the outer membrane of some gram-negative bacteria, conferring resistance to polymyxin. This resistance mechanism has resulted in a global public health issue due to the increased use of polymyxin as last-resort antibiotic treatments against multi-drug-resistant pathogens (Samantha and Vrielink, 2020). The phosphate-import ATP-binding protein is involved in phosphonates, phosphate esters, phosphite and phosphate import, responsible for energy coupling to the transport system (Junhong et al., 2023). Another enzyme phosphate-import ATP-binding protein belongs to a larger family that includes phosphate, phosphite, and phosphonate transporters, which binds strongly to inorganic phosphate (Feingersch et al., 2012). The transcriptional regulators SlyA are often involved in the regulation of genes important for bacterial virulence and stress response. However, the *slyA* deletion mutant (Δ*slyA*) of *Enterococcus faecalis* showed more virulence in an insect infection model (*Galleria mellonella*), exhibited increased persistence in mouse kidneys and liver, and survives better inside peritoneal macrophages (Michaux et al., 2011).

The 12^th^ cluster (Region 1.12) genes included efflux pump periplasmic linker (BepF) and membrane transporter (BepE), toluene efflux pump outer membrane protein (Ttg), Iron import ATP-binding/permease protein (IrtB & IrtA), D-alanine–D-alanyl carrier protein ligase, and regulatory protein (PchR) (**Supplementary Table 9**). Additionally, this region also possessed thioesterase (PikA5), isochorismate pyruvate lyase, salicylate biosynthesis isochorismate synthase, Inner membrane transport protein (YajR) and UvrABC system protein A. The UvrABC play critical role in DNA repair by nucleotide excision repair, replacing these aberrant nucleotides, involves the removal of twelve nucleotides where a genetic mutation has occurred followed by a DNA polymerase, and completing the DNA repair (Kraithong et al., 2021). Furthermore, this cluster possessed phenazine-1-carboxylate N-methyltransferase, which is involved in the biosynthesis of pyocyanin, a toxin produced and secreted by the *P. aeruginosa*, and plays a role in virulence (Mavrodi et al., 2001).

The 13^th^ cluster (Region 1.13) genes included pseudopaline exporter and synthase, metal-pseudopaline receptor CntO, biosynthetic arginine decarboxylase and putative Nudix hydrolase YfcD (**Supplementary Table 10**). The pseudopaline genes are involved in biosynthesis of metallophores, their export in the extracellular medium, and the recovery of a metal-metallophore complex under metal scarce conditions (Lhospice et al., 2017). The Nudix hydrolase contribute to cellular ‘housekeeping’ through the breakdown of a wide range of nucleoside diphosphate derivatives (Tong et al., 2009).

A number of secretion systems were identified in S-8 genome such as type II secretion systems (T2SS) that secrete the folded proteins such as pseudolysin (lasB), phospholipase C (PlcH), or lipase (LipA) from the periplasm to extracellular milieu (Costa et al., 2015). The presence of type III secretion system (T3SS) allows the translocation of bacterial effectors proteins into the host cell (de Bentzmann and Plesiat, 2011; Girlich et al., 2004), and was associated with bacterial persistence in the lungs and increased mortality in patients suffering from acute respiratory infections (Anantharajah, 2017). Type VI (T6SS) plays crucial role in bacterial competition and pathogenesis (Wood et al., 2019). The presence of these diverse secretion systems also facilitates the survival of S-8 under different environment niche.

The VFDB and CARD based analysis revealed various genes associated with antibiotic resistance and virulence activity of strain S-8. Notably, the presence of gene encoded β-lactamases enzyme *bla*_PDC-142_ and *bla*_PME-1_ is consistent with carbapenem resistance. Additionally, the following cephalosporin-resistant genes, *bla*_PDC-2_, *bla*_PDC-7_, and *bla*_PDC-9_ was observed and one each for aminoglycoside (*aph*(*3*′)*-IIb*) fosfomycin (*fosA*) and chloramphenicol (*catB7*) resistance genes was present. The gene *arnA* modifies the lipid A with 4-amino-4-deoxy-l-arabinose (Ara4N), which allows Gram-negative bacteria to resist antimicrobial peptides and antibiotics such as polymyxin. A previous study (Udaondo et al., 2016) reported that *P. putida* strains that share 85% of the coding regions with *P. aeruginosa* bear the various genes encoded for transporters, enzymes and regulators for amino acid metabolism and reveals their environmental applications by the capacity to degrade pollutants and ability to promote plant growth. This research represents an effort to find the mechanisms underlying the ecology, pathogenicity and evolution history of *Pseudomonas* spp. that able to develop biotechnological advances. Cho et al., 2015 reported *P. fluorescens* PCL1751 genome and comparative genome study that reveals the integration of prophages that play an important role in genome rearrangements and this strain achieves biological control of pathogens through effective competition for nutrients including niches.

Exposure to metal stressors significantly inhibits the respiratory enzymatic activities as well as corresponding transcript level (Samanta et al., 2020). In S-8 genome, a large inventory of CAZymes was noted including sugar metabolizing enzymes, which confers respiratory metabolism under metal stressors, facilitating the high energy metabolism activity. *P. aeruginosa* shows a large degree of genomic heterogeneity both through variation in sequences found throughout the species (core genome) and through the presence or absence of sequences in different isolates (accessory genome) (Pincus et al., 2020). *P. aeruginosa* isolates also differ markedly in their ability to cause disease. In this study, we investigated the genomic features of a newly isolated environmental strain *P. aeruginosa* S-8. We showed that core genome, alone or in combination with the accessory genome are also predictive of virulence. Another interesting genomic trait, the presence of several GIs was observed in S-8 genome. In bacteria, these GIs are decorated with additional genes acquired via horizontal gene transfer (HGT) mechanism. The presence of these genes may render additional metabolic functions including adaptive traits and genome plasticity which may facilitate evolutionary survival (Pérez-Pantoja et al., 2013). Recent genomic investigation has revealed GIs encoded functional traits which are classified into PAIs (pathogenicity islands) encoding virulence genes, MIs (metabolic islands) encoding biosynthesis of secondary metabolites, RIs (resistance islands) encoding resistance genes to antibiotics, and SIs (symbiotic islands) encoding genes for symbiotic association of the host to microorganism. The observed results suggest a strong possibility that in S-8 strain GIs were recruited via HGT to facilitate the survival in different environmental conditions.

The microbial genome variation is essential for understanding microbial functions, microbe-host interactions, and to understand the effects of genetic variation on function or phenotype. The small genomic differences may influence the phenotype provides evidence about the functional consequence of sequence variation. Over decades, microbiologists have identified the function of numerous genes across multiple species mainly through investigating the effect of gene loss. The diversity of functional genetic features may greatly exceed the taxonomic diversity due to horizontal gene transfer (HGT) and rapid evolutionary adaptation (Wiedenbeck and Cohan, 2011). The BGCs clusters of S-8 strains showed both strain-specific and unidentified characteristics, which support the idea that bacteria perform metabolic activity exclusively for survival in a particular ecological environment and potentially construct alternative routes for new bioactive metabolite production. The test isolate S-8 showed a higher number of virulence genes as compared to other tested genomes, therefore, genome analysis of S-8 reveals the number of potential features that might be considered candidates for future studies to explore the virulence mechanism deployed by this bacterium. *P. aeruginosa* enriched with conserved core genome of low sequence diversity and variable genome components that communicate with other *Pseudomonas* by horizontal gene transfer (Klockgether et al., 2011).

## Conclusion

In summary, several genomic features of *P. aeruginosa* S-8 were identified based on the whole-genome analysis. Phylogenetic based analysis showed that strain S-8 has a high similarity to *P. aeruginosa* strains. Comparative genomic analysis revealed that S-8 possesses genomic islands, prophages, etc. We identified some putative virulence factors and future studies should expand the number of isolates, so as to increase the confidence of results generated in the present study. The ability of the genome to predict antibiotic resistance genes opens the door for sequencing of new strains and it will further supplement or replace the traditional antimicrobial susceptibility testing. However, future studies are needed to provide detailed understandings of the role that genetic variation plays the ability of *P. aeruginosa* to cause disease by using suitable model system. By being aware of the potentially high virulence of the organisms, personal safety measurements can be increased to avoid an accidental exposition of the organism. The presence of various CAZymes illustrate its importance in industry regarding complex polysaccharide degradation and further energy production. Using newly sequenced data and their investigation can help to substantially speed up research in the future and to draw wider, more general conclusions.

## Data Availability

The datasets presented in this study can be found in online repositories. The names of the repository/repositories and accession number(s) can be found in the article/**Supplementary Material**.

## Supplementary material

Supplementary Figure 1 Phylogenetic analysis based on 16S rRNA confirmed that S-8 belongs to *P. aeruginosa.* The 16s rRNA gene was amplified and sequenced. The obtained sequence was aligned using CLUSTAL-X and tree was constructed using neighbor-joining (NJ) method with bootstraps of 1000 replicates.

Supplementary Figure 2 Antagonistic activity of *P. aeruginosa* S-8 against bacterial pathogen *E. coli* **(A)**, *S. typhi* **(B)**, and fungal strain *M. gypsium.*

Supplementary Figure 3 To re-evaluate the phylogenetic relationship of S-8, ANI analysis was calculated with respect to other sequenced *P. aeruginosa* strains.

Supplementary Figure 4 The fastANI analyses confirmed the closest similarity of S-8 to *P. aeruginosa* F30658 and *P. aeruginosa* W16407.

Supplementary Figure 5
**(A)** OrthoVenn diagram showing the number of common and separate protein clusters for S-8 and other closely related genomes, the occurrence table contains groups of gene clusters like cluster count and protein count. Row indicates the orthologous gene cluster for multiple species that summarized as a cell graph and column indicates different closely related bacterial species, **(B)** The pairwise protein sequence comparison for heatmap showing orthologous clusters between S-8 and other closely related strains.

Supplementary Figure 6
**(A)** The pan-genome and core-genome analysis was performed using the Box-plot analysis which showed that among the selected strains, S-8 showed the less conserved genes, and a high number of unique genes (Supplementary Figure 6B).
