# Supplementary figures and images for: In-depth genome and comparative genome analysis of a metal-resistant environmental isolate Pseudomonas aeruginosa S-8
Source: Front Cell Infect Microbiol. 2025 Feb 27;15:1511507. doi: 10.3389/fcimb.2025.1511507 (PMC11903748; doi:10.3389/fcimb.2025.1511507)

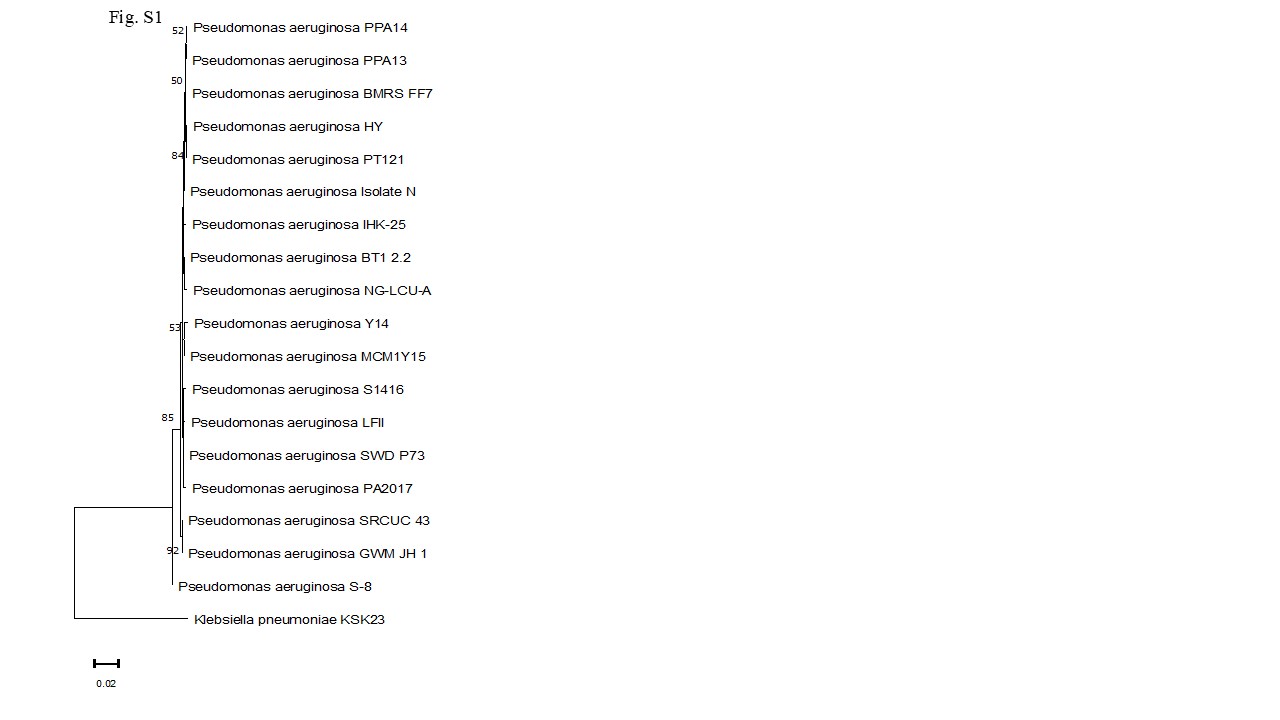

Supplement: Supplementary Figure 1 — Phylogenetic analysis based on 16S rRNA confirmed that S-8 belongs to P. aeruginosa. The 16s rRNA gene was amplified and sequenced. The obtained sequence was aligned using CLUSTAL-X and tree was constructed using neighbor-joining (NJ) method with bootstraps of 1000 replicates. [file Image1.jpeg]

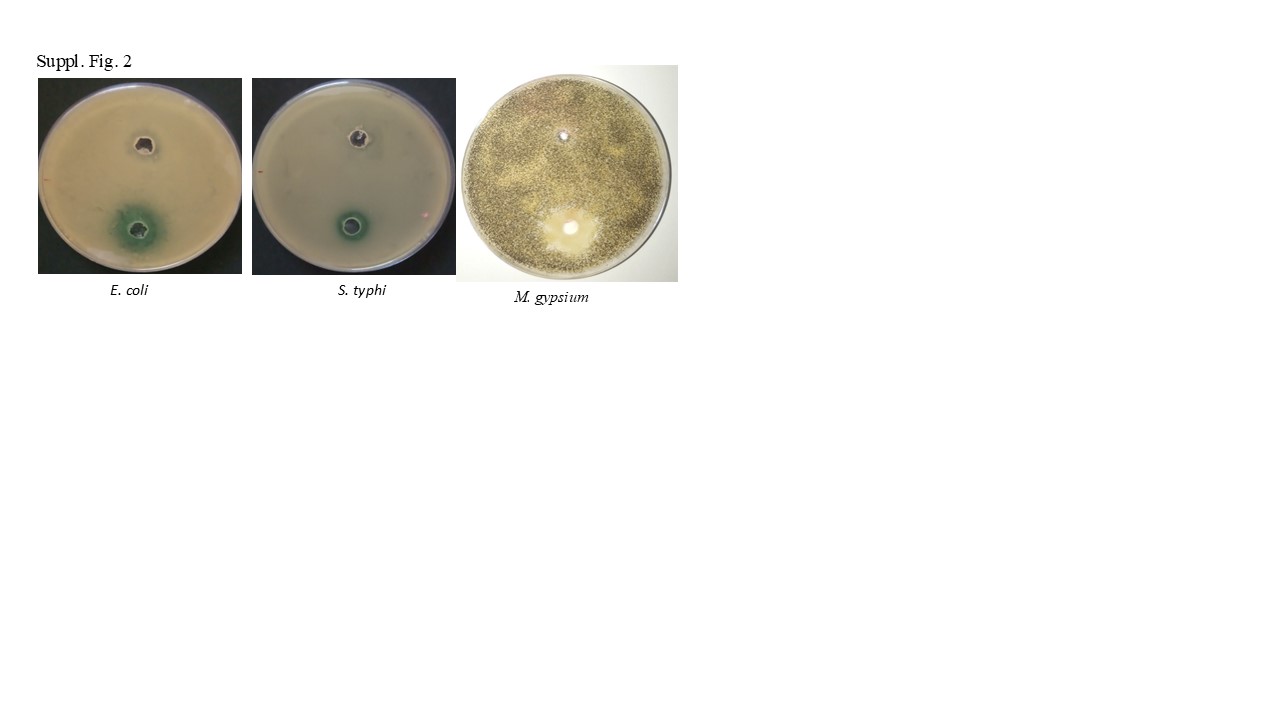

Supplement: Supplementary Figure 2 — Antagonistic activity of P. aeruginosa S-8 against bacterial pathogen E. coli (A), S. typhi (B), and fungal strain M. gypsium. [file Image2.jpeg]

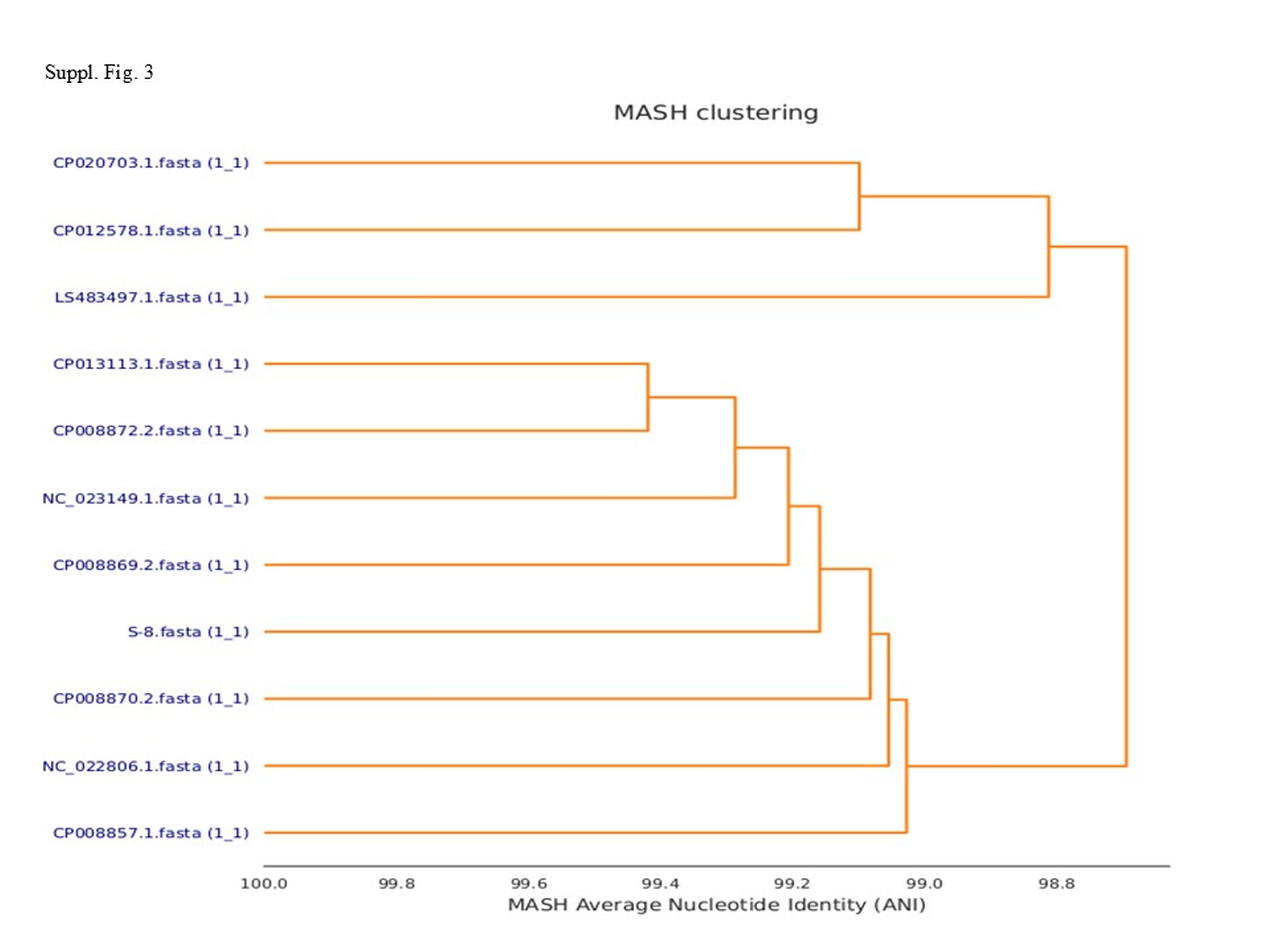

Supplement: Supplementary Figure 3 — To re-evaluate the phylogenetic relationship of S-8, ANI analysis was calculated with respect to other sequenced P. aeruginosa strains. [file Image3.jpeg]

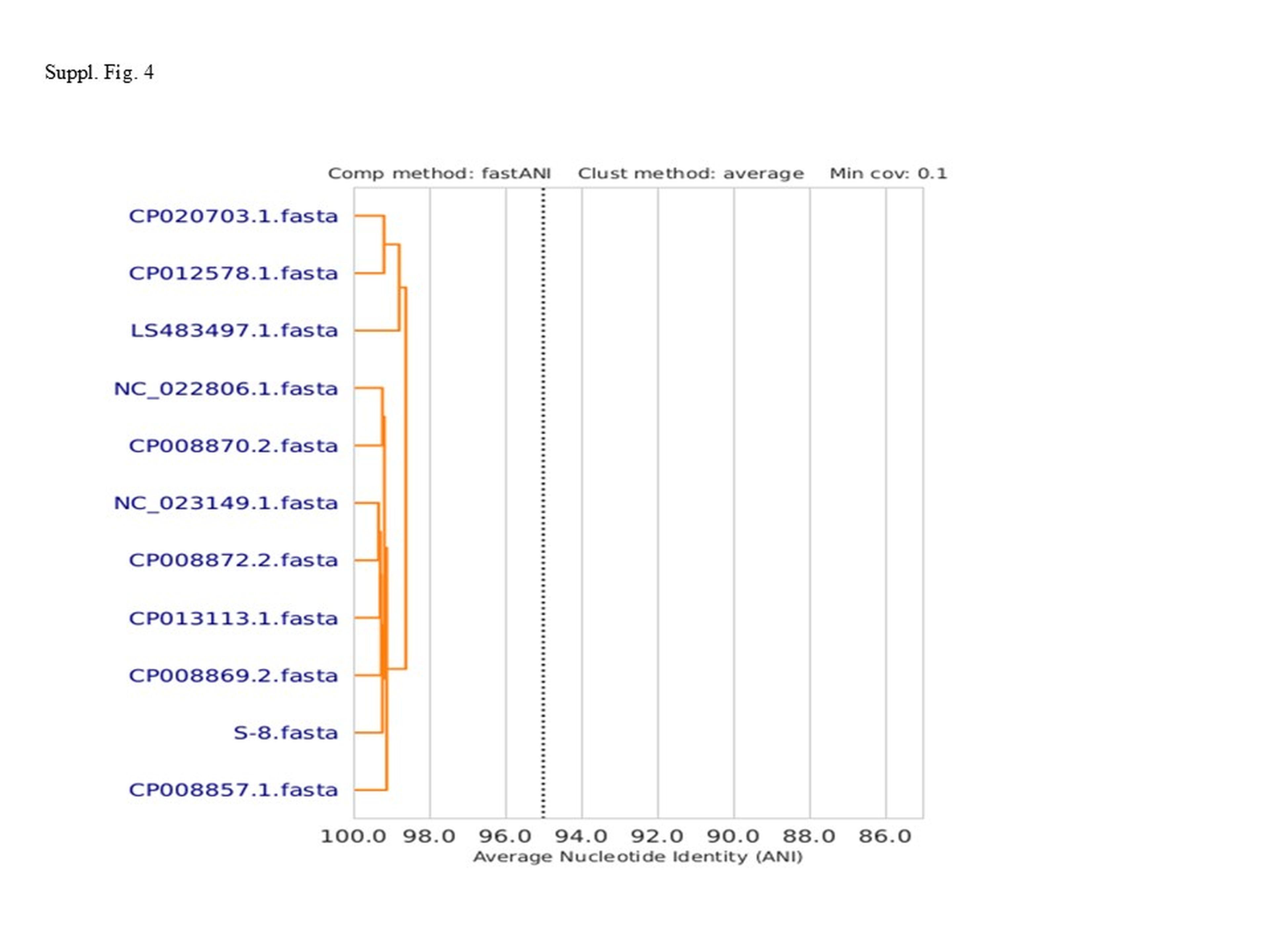

Supplement: Supplementary Figure 4 — The fastANI analyses confirmed the closest similarity of S-8 to P. aeruginosa F30658 and P. aeruginosa W16407. [file Image4.jpeg]

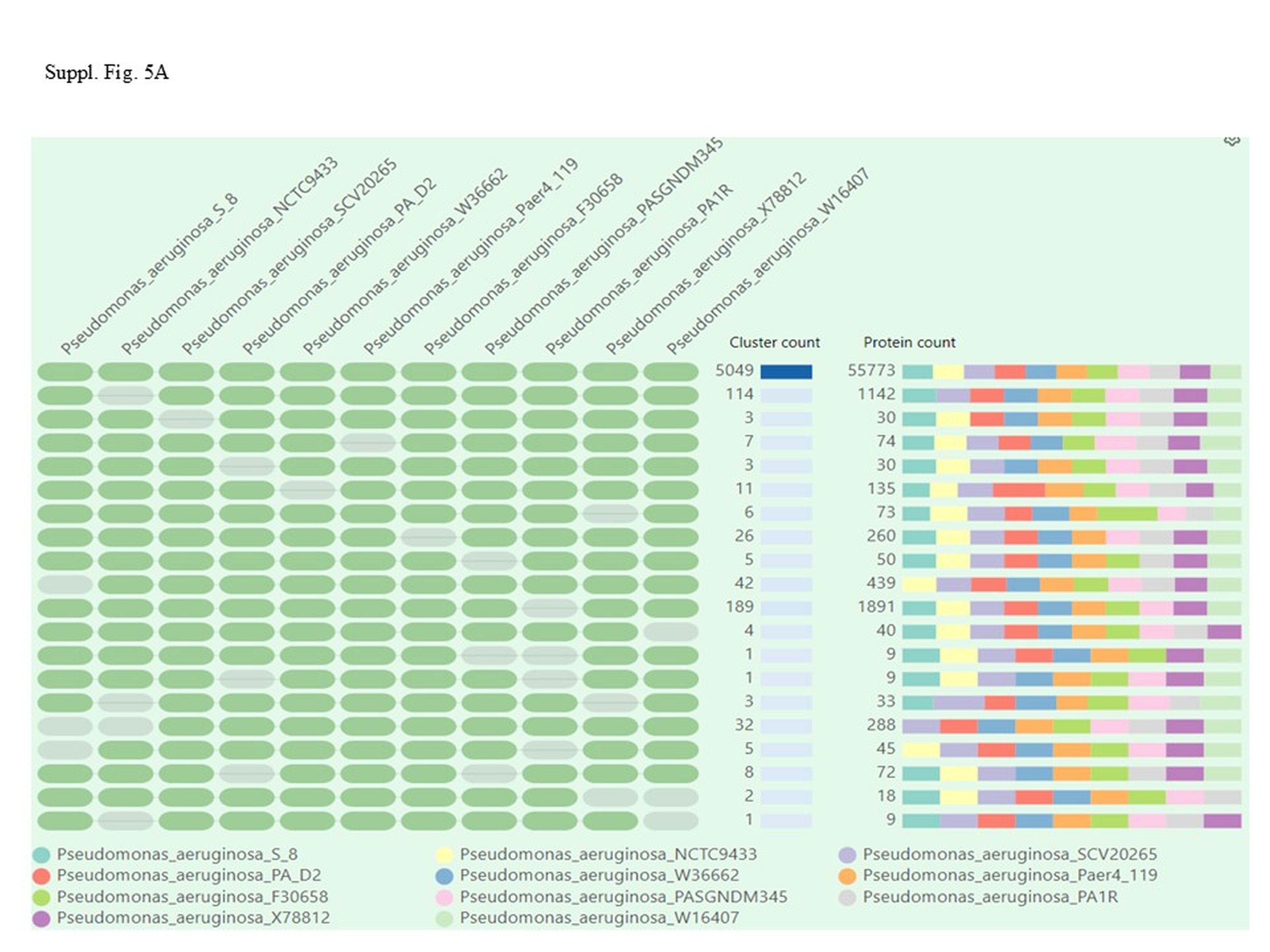

Supplement: Supplementary Figure 5 — (A) OrthoVenn diagram showing the number of common and separate protein clusters for S-8 and other closely related genomes, the occurrence table contains groups of gene clusters like cluster count and protein count. Row indicates the orthologous gene cluster for multiple species that summarized as a cell graph and column indicates different closely related bacterial species, (B) The pairwise protein sequence comparison for heatmap showing orthologous clusters between S-8 and other closely related strains. [file Image5.jpeg]

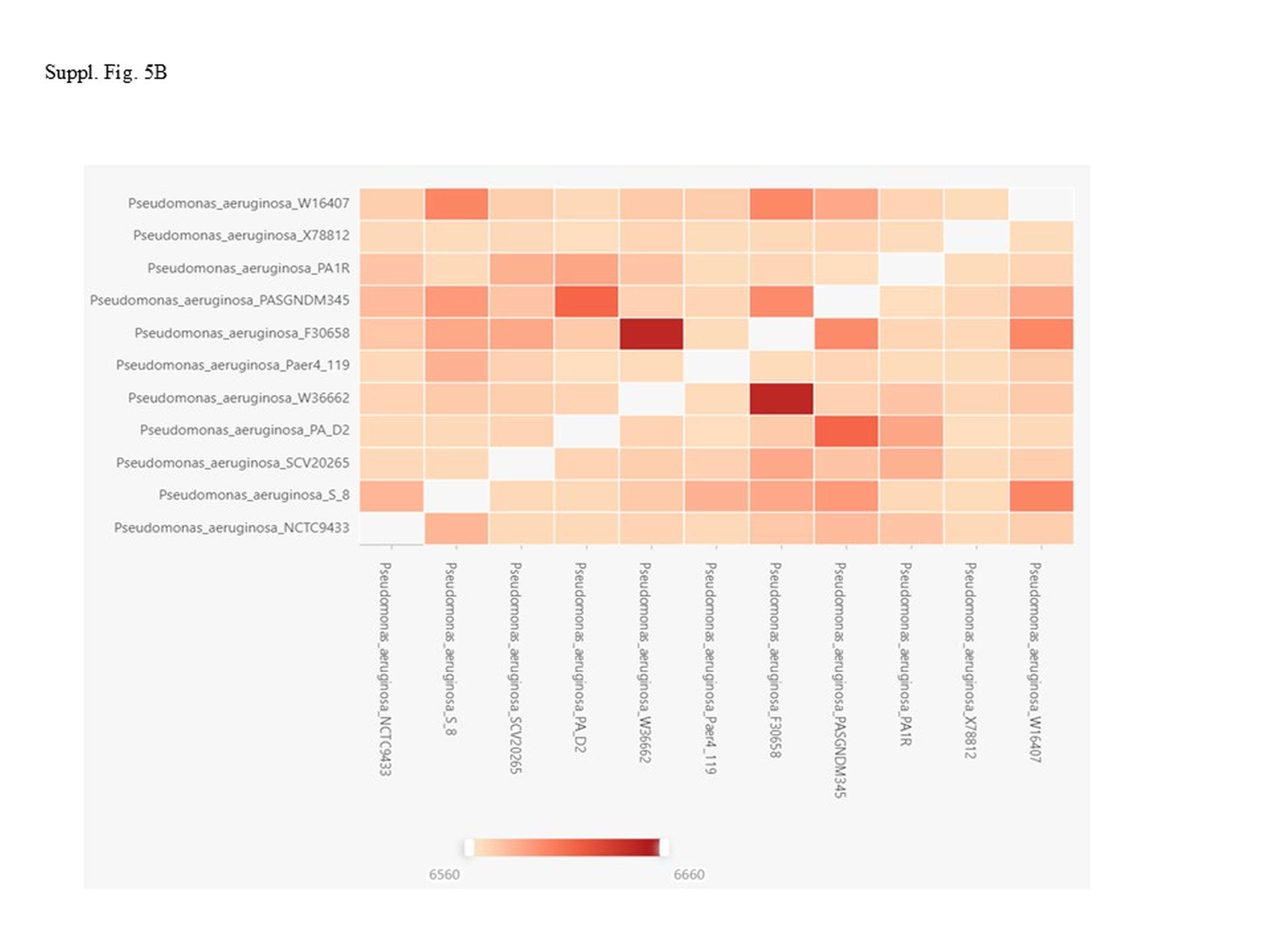

Supplement: Supplementary Figure 6 — (A) The pan-genome and core-genome analysis was performed using the Box-plot analysis which showed that among the selected strains, S-8 showed the less conserved genes, and a high number of unique genes (Supplementary Figure 6B). [file Image6.jpeg]

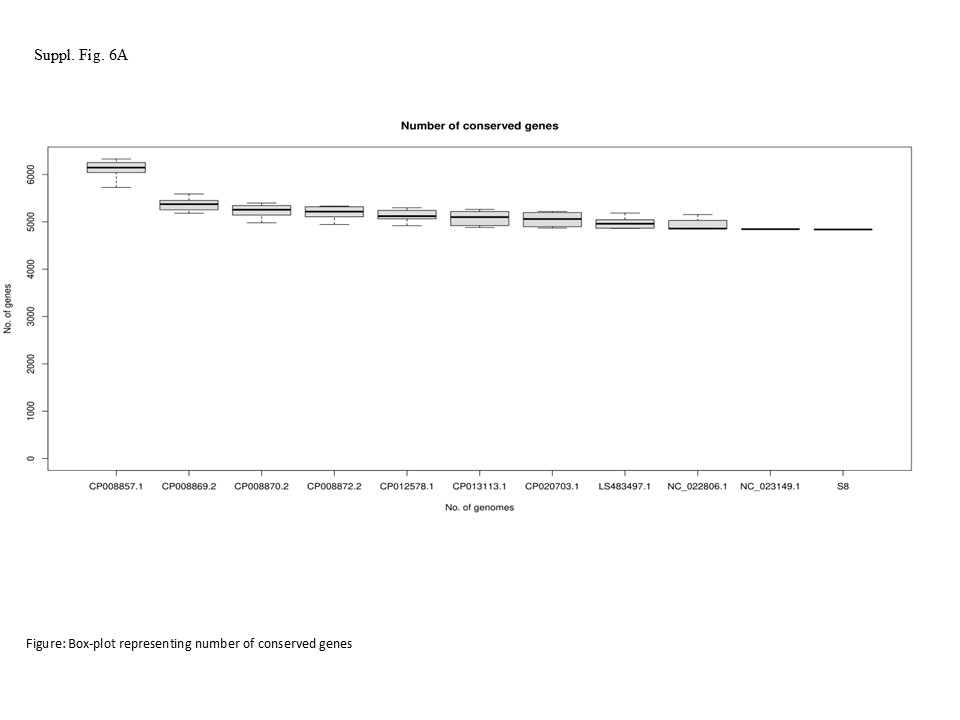

Supplement: Supplementary file 7 [file Image7.jpeg]

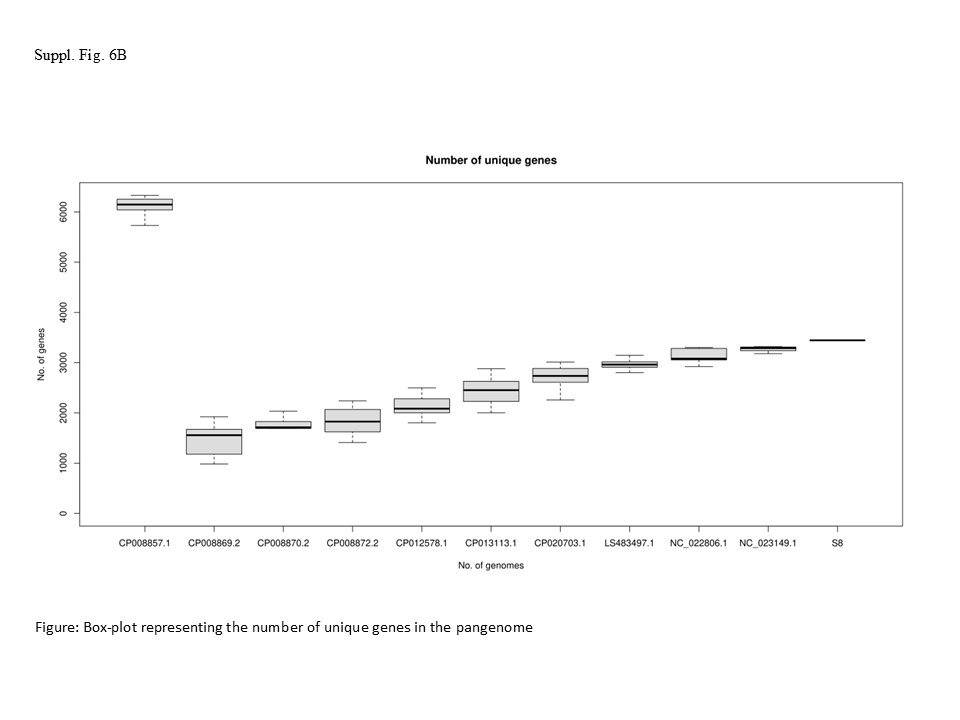

Supplement: Supplementary file 8 [file Image8.jpeg]
