# Supplementary material for: In-depth genome and comparative genome analysis of a metal-resistant environmental isolate Pseudomonas aeruginosa S-8
Source: Front Cell Infect Microbiol. 2025 Feb 27;15:1511507. doi: 10.3389/fcimb.2025.1511507 (PMC11903748; doi:10.3389/fcimb.2025.1511507)
Supplement: Supplementary file 9 [file Table1.docx]

**Supplementary Table 6** List of secondary metabolite genes in region 1.*5*

| GENE NAME | LOCATION FROM | TO | ANNOTATION OF QUERY CLUSTER |
| --- | --- | --- | --- |
|  |  |  |  |
| EEEKKGKG_02378 | 2,509,979 | 2,512,111 | Vitamin B12 transporter BtuB |
| EEEKKGKG_02382 | 2,516,874 | 2,517,728 | Nitrate import ATP-binding protein NrtD |
| EEEKKGKG_02387 | 2,522,140 | 2,522,889 | HTH-type transcriptional repressor NagR |
| EEEKKGKG_02390 | 2,525,346 | 2,531,723 | D-alanine--D-alanyl carrier protein ligase |
| EEEKKGKG_02393 | 2,533,877 | 2,537,626 | Linear gramicidin synthase subunit B |
| EEEKKGKG_02394 | 2,537,722 | 2,538,339 | Cysteine/O-acetylserine efflux protein |
| EEEKKGKG_02396 | 2,539,332 | 2,540,180 | Bicarbonate transport ATP-binding protein CmpD |
| EEEKKGKG_02398 | 2,541,251 | 2,542,138 | Alpha-ketoglutarate-dependent taurine dioxygenase |
| EEEKKGKG_02402 | 2,544,092 | 2,545,345 | Purine ribonucleoside efflux pump NepI |
| EEEKKGKG_02403 | 2,545,345 | 2,546,520 | Esterase EstB |
| EEEKKGKG_02404 | 2,546,635 | 2,547,528 | HTH-type transcriptional regulator PgrR |
| EEEKKGKG_02405 | 2,547,632 | 2,548,930 | Gamma-glutamyl putrescine oxidoreductase |
| EEEKKGKG_02407 | 2,549,916 | 2,550,947 | HTH-type transcriptional regulator GntR |
| EEEKKGKG_02411 | 2,553,445 | 2,555,070 | NADP-dependent glyceraldehyde-3-phosphate dehydrogenase |
| EEEKKGKG_02412 | 2,555,288 | 2,556,547 | putative FMNH2-dependent monooxygenase SfnC |

**Supplementary Table 7** List of secondary metabolite genes in region 1.*7*

| GENE NAME | LOCATION FROM | TO | ANNOTATION OF QUERY CLUSTER |
| --- | --- | --- | --- |
|  |  |  |  |
| **EEEKKGKG_02677** | 2,906,620 | 2,907,834 | L-lactate transporter |
| **EEEKKGKG_02679** | 2,910,619 | 2,911,425 | HTH-type transcriptional regulator MalT |
| **EEEKKGKG_02678** | 2,907,845 | 2,910,499 | Vitamin B12 transporter BtuB |
| **EEEKKGKG_02683** | 2,914,163 | 2,915,671 | Antibiotic efflux pump outer membrane protein ArpC |
| **EEEKKGKG_02684** | 2,915,671 | 2,916,789 | putative multidrug ABC transporter permease YbhR |
| **EEEKKGKG_02685** | 2,916,794 | 2,917,927 | putative multidrug ABC transporter permease YbhS |
| **EEEKKGKG_02686** | 2,917,940 | 2,919,709 | putative multidrug ABC transporter ATP-binding protein YbhF |
| **EEEKKGKG_02689** | 2,921,487 | 2,922,281 | Linear gramicidin dehydrogenase LgrE |
| **EEEKKGKG_02690** | 2,922,285 | 2,923,778 | putative L-prolyl-AMP ligase PigI |
| **EEEKKGKG_02691** | 2,923,775 | 2,924,908 | L-prolyl-[peptidyl-carrier protein] dehydrogenase |
| **EEEKKGKG_02692** | 2,924,910 | 2,926,547 | Tetracycline 7-halogenase |
| **EEEKKGKG_02697** | 2,941,539 | 2,942,417 | HTH-type transcriptional regulator DmlR |
| **EEEKKGKG_02699** | 2,944,054 | 2,945,103 | 1,3,6,8-tetrahydroxynaphthalene synthase |
| **EEEKKGKG_02703** | 2,948,431 | 2,949,582 | Putative acyl-CoA dehydrogenase YdbM |
| **EEEKKGKG_02704** | 2,949,587 | 2,950,669, | Methanesulfonate monooxygenase |
| **EEEKKGKG_02706** | 2,951,633 | 2,952,721 | Alkanesulfonate monooxygenase |
| **EEEKKGKG_02707** | 2,953,040 | 2,953,936 | HTH-type transcriptional regulator YofA |
| **EEEKKGKG_02709** | 2,954,651 | 2,956,234 | Thiosulfate sulfurtransferase GlpE |
| **EEEKKGKG_02717** | 2,959,822 | 2,960,796 | Glutathionyl-hydroquinone re |
| **EEEKKGKG_02718** | 2,960,801 | 2,962,198 | Siroheme synthase |

**Supplementary Table 8** List of secondary metabolite genes in region 1.*8*

| GENE NAME | LOCATION FROM | TO | ANNOTATION OF QUERY CLUSTER |
| --- | --- | --- | --- |
| EEEKKGKG_03544 | 3,784,399 | 3,786,054 | Phosphoethanolamine transferase EptA |
| EEEKKGKG_03545 | 3,786,381 | 3,786,381 | putative signaling protein |
| EEEKKGKG_03547 | 3,789,604 | 3,790,461 | putative ABC transporter phosphite binding protein PhnD1 |
| EEEKKGKG_03548 | 3,790,458 | 3,791,261 | Phosphate-import ATP-binding protein PhnC |
| EEEKKGKG_03556 | 3,797,176 | 3,798,090 | PCP degradation transcriptional activation protein |
| EEEKKGKG_03559 | 3,800,094 | 3,801,872 | putative oxidoreductase EphD |
| EEEKKGKG_03562 | 3,804,200 | 3,811,258 | Dimodularnon ribosomal peptide synthase |
| EEEKKGKG_03563 | 3,811,255 | 3,812,421 | Aurachin C monooxygenase/isomerase |
| EEEKKGKG_03566 | 3,814,655 | 3,815,911 | Cytochrome P450 107B1 |
| EEEKKGKG_03568 | 3,816,337 | 3,817,329 | 3-oxoacyl-[acyl-carrier-protein] synthase 3 |
| EEEKKGKG_03569 | 3,817,343 | 3,817,343 | Acyl carrier protein |
| EEEKKGKG_03571 | 3,818,544 | 3,819,710 | Inner membrane transport protein YdhP |
| EEEKKGKG_03572 | 3,819,721 | 3,820,713 | ADP-L-glycero-D-manno-heptose-6-epimerase |
| EEEKKGKG_03576 | 3,825,687 | 3,826,121 | Transcriptional regulator SlyA |
| EEEKKGKG_03579 | 3,828,664 | 3,830,802 | ATP-dependent DNA helicase RecQ |
